# Postmortem Skeletal Microbial Community Composition and Function in Buried Human Remains

**DOI:** 10.1128/msystems.00041-22

**Published:** 2022-03-30

**Authors:** Alexandra L. Emmons, Amy Z. Mundorff, Katharina M. Hoeland, Jonathan Davoren, Sarah W. Keenan, David O. Carter, Shawn R. Campagna, Jennifer M. DeBruyn

**Affiliations:** a Department of Anthropology, University of Tennessee, Knoxville, Tennessee, USA; b Department of Chemistry, University of Tennessee, Knoxville, Tennessee, USA; c Bode Cellmark Forensics, Lorton, Virginia, USA; d Department of Geology and Geological Engineering, South Dakota School of Mines and Technology, Rapid City, South Dakota, USA; e School of Natural Sciences and Mathematics, Chaminade University of Honolulu, Honolulu, Hawaii, USA; f Department of Biosystems Engineering and Soil Science, University of Tennessee, Knoxville, Tennessee, USA; University of Illinois at Urbana-Champaign

**Keywords:** subsurface decomposition, skeletal DNA, microbial ecology, microbiome, metabolomics, bone, skeleton, soil microbiology, taphonomy

## Abstract

Bones and teeth can provide a lasting resource to identify human remains following decomposition. Bone can support dynamic communities of micro- and macroscopic scavengers and incidental taxa, which influence the preservation of bone over time. Previously we identified key microbial taxa associated with survivability of DNA in bones of surface-decomposed human remains, observing high intra- and interindividual variation. Here we characterized the postmortem bone microbiome of skeletal remains in a multi-individual burial to better understand subsurface bone colonization and preservation. To understand microbial community origins and assembly, 16S rRNA amplicon sequences from 256 bone and 27 soil samples were compared to bone from individuals who decomposed on the ground surface, and human gut sequences from the American Gut Project. Untargeted metabolomics was applied to a subset of 41 bone samples from buried remains to examine potential microbe–metabolite interactions and infer differences related to community functionality. Results show that postmortem bone microbial communities are distinct from those of the oxic surface soils and the human gut. Microbial communities from surface-deposited bone and shallow buried bone were more similar to those from soils, while bones recovered from saturated areas deeper in the grave showed increased similarity with human gut samples with higher representation of anaerobic taxa, suggesting that the depositional environment affected the established bone microbiome. Correlations between metabolites and microbes indicate that phosphate solubilization is likely an important mechanism of microbially mediated skeletal degradation. This research expands our knowledge of microbial bone colonizers, including colonizers important in a burial environment.

**IMPORTANCE** Understanding the microbes that colonize and degrade bone has important implications for preservation of skeletal elements and identification of unknown human remains. Current research on the postmortem bone microbiome is limited and largely focuses on archaeological or marine contexts. Our research expands our understanding of bone microbiomes in buried remains by characterizing the taxonomic and metabolic diversity of microbes that are colonizing bone after a 4-year postmortem burial interval and examines the potential impact of microbial colonization on human skeletal DNA preservation. Our results indicate that the postmortem bone microbiome is distinct from the human gut and soil. Evidence from combined metabolomic and amplicon sequencing analysis suggests that Pseudomonas and phosphate solubilization likely play a role in skeletal degradation. This work provides important insight into the types and activities of microbes controlling the preservation of buried skeletal remains.

## INTRODUCTION

After an individual dies, soft tissue and identifiable physical characteristics decompose leaving only bones and teeth. From a forensic perspective, bones and teeth provide a critical resource for identifying unknown individuals, whether by offering important clues about a decedent’s sex, stature, or estimated age at death (1) or through a direct identification by way of skeletal DNA (2). Skeletal preservation is influenced by bone diagenetic processes, including chemical and/or biological modification (3). Soil geochemistry and site hydrology are important to bone preservation and influence microbial degradation (3, 4), including the diversity and abundance of microbes present in a given environment. Dissolution and recrystallization are affected by moisture availability, pH, and bone porosity (4–6). Following autolysis, pore spaces once occupied by soft tissues in living bone become accessible to water, ionic exchange (4, 6), and microorganisms (5). The dissolution and recrystallization of bioapatite, the primary mineral phase in bone, is important to the preservation of organic components of bone including collagen and DNA (4, 5, 7). As bioapatite dissolves it releases bound DNA and exposes collagen to hydrolytic enzyme activity (7, 8) via microbial collagenases (5). Along with water and pH, microbes can increase the rate of dissolution and effectively degrade bone from the outside-in through the production of organic acids (9) and chelating agents (10).

When individuals decompose on the ground surface, human remains are exposed to more variable environmental conditions including changes in temperature, humidity, precipitation, arthropod activity, UV exposure, and scavenging (11, 12), which would influence microbial community structure. In fact, in surface remains, Deinococcus-Thermus, a group composed of extremophiles, was observed in greater than 2% relative abundance in cranial elements, likely related to the duration of exposure (13). The suite of variables affecting decomposition in burials are different than those that apply to surface decomposition. Subsurface environments are influenced by site hydrology, soil composition, moisture, burial depth, microbial ecology, and other edaphic parameters (14, 15). Unlike surface sites, burials mostly preclude access by scavengers and insects and minimize temperature effects (16, 17).

Previously, we found that bone microbial communities in surface remains had considerable intra-individual variation and exhibited clustering by anatomical region (13). We hypothesized that this was due to the uniqueness of individual microbiomes in life, the physical distance between bones and the gut, and the variability in environmental conditions after death (13). Because factors modulating the rate and trajectory of decomposition differ between buried and surface environments, and putrefactive bacteria have been implicated in bone degradation (18–20) and are more likely to persist under anoxic conditions, we expected the state of decay of buried remains to be more dependent on microbial community structure than surface remains. Thus, we expect microbial communities from buried bone to show differences from surface bone related to changes in the deposition environment.

The primary goal of this study was to examine differences in microbial community structure in skeletal remains from individuals who decomposed on the ground surface compared to bones from individuals who decomposed within a burial, to better characterize the postmortem bone microbiome. Because we used microbial DNA co-extracted with human DNA, a secondary goal was to relate microbial community changes to human skeletal DNA preservation in bone to better understand the impact of microbes on skeletal integrity and DNA preservation. Additionally, soil samples collected from within and outside of the grave and gut samples from the American Gut Project (AGP) were used to relate bone microbial communities to their potential sources, i.e., local environmental and enteric microorganisms. Lastly, untargeted metabolomics was applied to a subset of bone samples to potentially infer functional attributes related to microbial community differences within a multi-individual human grave.

## RESULTS

### Bone and soil microbial community composition.

Rarefaction curves indicate that our sequencing depth was adequate for the majority of samples (Fig. S1 in the supplemental material). Thirty-nine bacterial and archaeal phyla were identified in buried bone samples. Dominant taxa (mean relative abundance > 2%) included Proteobacteria (30.1–75.1%), Firmicutes (1.9–41.1%), Actinobacteria (1.7–23.8%), and Bacteroidetes (3.8–20.7%). This was comparable to 37 phyla identified in surface bones, with dominant representatives from Proteobacteria (21.1–56.2%), Actinobacteria (2.27–35.2%), Bacteroidetes (4.8–25.6%), Firmicutes (2.3–35.2%), Patescibacteria (0.27–13.6%), and Planctomycetes (0.09–9.7%). A total of 36 phyla were observed in soil samples, including controls and off-grave samples. Dominant taxa in soil included Proteobacteria (19.6–58.5%), Actinobacteria (10.6–33.0%), Verrucomicrobia (0.9–30.1%), Acidobacteria (0.7–20.6%), Chloroflexi (1.8–7.5%), Firmicutes (0.3–17.9%), Bacteroidetes (1.4 −10.1%), Thaumarchaeota (0.04–5.9%), and Planctomycetes (0.4–3.6%) (Fig. S2).

10.1128/msystems.00041-22.6 FIG S1 Rarefaction curves with a sequencing depth of 10,000. Facets represent individuals (SA, SB, SC, A, B, C) or sample type (soil and AGP). Each line represents a sample. Species richness refers to observed OTUs. Download FIG S1, TIF file, 9.6 MB. Copyright © 2022 Emmons et al. 2022 Emmons et al. https://creativecommons.org/licenses/by/4.0/ This content is distributed under the terms of the Creative Commons Attribution 4.0 International license.

10.1128/msystems.00041-22.7 FIG S2 Bone bacterial and archaeal community composition. Samples were averaged by bone type and soil type. Soil samples include those collected with depth in the grave (“Grave”; depths = 0, 30, 40, 70), those collected 0.5 m off grave (“OG0.5 m”), and control soils, disturbed and undisturbed (“ControlG,” “ControlS”). All samples with less than 7,000 reads were filtered from the data set prior to visualization; data set was not rarefied. “Rare taxa” includes taxa with average relative abundances less than 1% evaluated by sample. Download FIG S2, TIF file, 14.7 MB. Copyright © 2022 Emmons et al. 2022 Emmons et al. https://creativecommons.org/licenses/by/4.0/ This content is distributed under the terms of the Creative Commons Attribution 4.0 International license.

### Alpha diversity.

Samples from the AGP, on average, had the lowest bacterial diversity and richness estimates, while soils had the greatest (Table S4). Bone samples showed intermediate species diversity and richness. Differences by sample type (bone, gut, soil) were statistically significant (Richness: Χ^2^ = 125.6, DF = 2, *P* < 2.2e-16; Inverse Simpson: Χ^2^ = 84.8, DF = 2, *P* < 2.2e-16). Although richness did not significantly differ by bone depositional environment (surface versus buried), mean bacterial diversity in surface bone (mean = 45.4) was greater than mean diversity in buried bone (mean = 28.9) (Χ^2^ = 29.1, DF = 1, *P* = 6.76e-08). Richness was significantly different between individuals (Χ^2^ = 146.9, DF = 5, *P* < 2.2e-16). Samples from individual C, which was the shallowest in the grave (closest to the ground surface), had the greatest mean species richness (all comparisons *P* < 1.0e-5, includes surface individuals), while individual A, located at the deepest point in the grave (base of grave), had the lowest (all comparisons *P* < 0.001). Individual B was located between A and C. Individuals A and B had similar and significantly lower diversity (inverse Simpson) compared to all other individuals (individual C and the surface remains; all comparisons *P* < 0.001). Bacterial diversity estimates from buried individual C were most similar to surface individuals SB and SC and had significantly greater diversity estimates than buried individuals A and B (*P* < 1.0e-10). Microbial species diversity and richness were variable by bone element type, but some trends did present. For example, bones of the lower and upper torso of individual A had some of the lowest richness and diversity estimates, while bones from the skull and foot had some of the highest. Arm bones had the lowest richness and diversity estimates in individuals B and C, while lower torso bones had the highest richness and diversity estimates in these same individuals (Fig. S3). Though mean diversity estimates from foot bones were intermediate to other anatomical regions, mean richness estimates were ranked second highest after the lower torso in individuals B and C (results not shown).

10.1128/msystems.00041-22.4 TABLE S4 Alpha diversity metrics by individual (A, B, C, SA, SB, SC) and sample type (AmerGut and Soil). Download Table S4, DOCX file, 0.01 MB. Copyright © 2022 Emmons et al. 2022 Emmons et al. https://creativecommons.org/licenses/by/4.0/ This content is distributed under the terms of the Creative Commons Attribution 4.0 International license.

10.1128/msystems.00041-22.8 FIG S3 Alpha diversity estimates by individual or sample type. Box and whisker plots made using ggplot2 and geom_box(). Facet labels A, B, C, SA, SB, and SC refer to bone samples from individuals combined by anatomical region. Soil samples are either from control soils (ControlS), off grave soils at 0.5 m (OG0.5 m), or grave soil samples (Grave). Download FIG S3, TIF file, 10.5 MB. Copyright © 2022 Emmons et al. 2022 Emmons et al. https://creativecommons.org/licenses/by/4.0/ This content is distributed under the terms of the Creative Commons Attribution 4.0 International license.

### Beta diversity: sample type, individual, and anatomical region.

**FIG 1:**
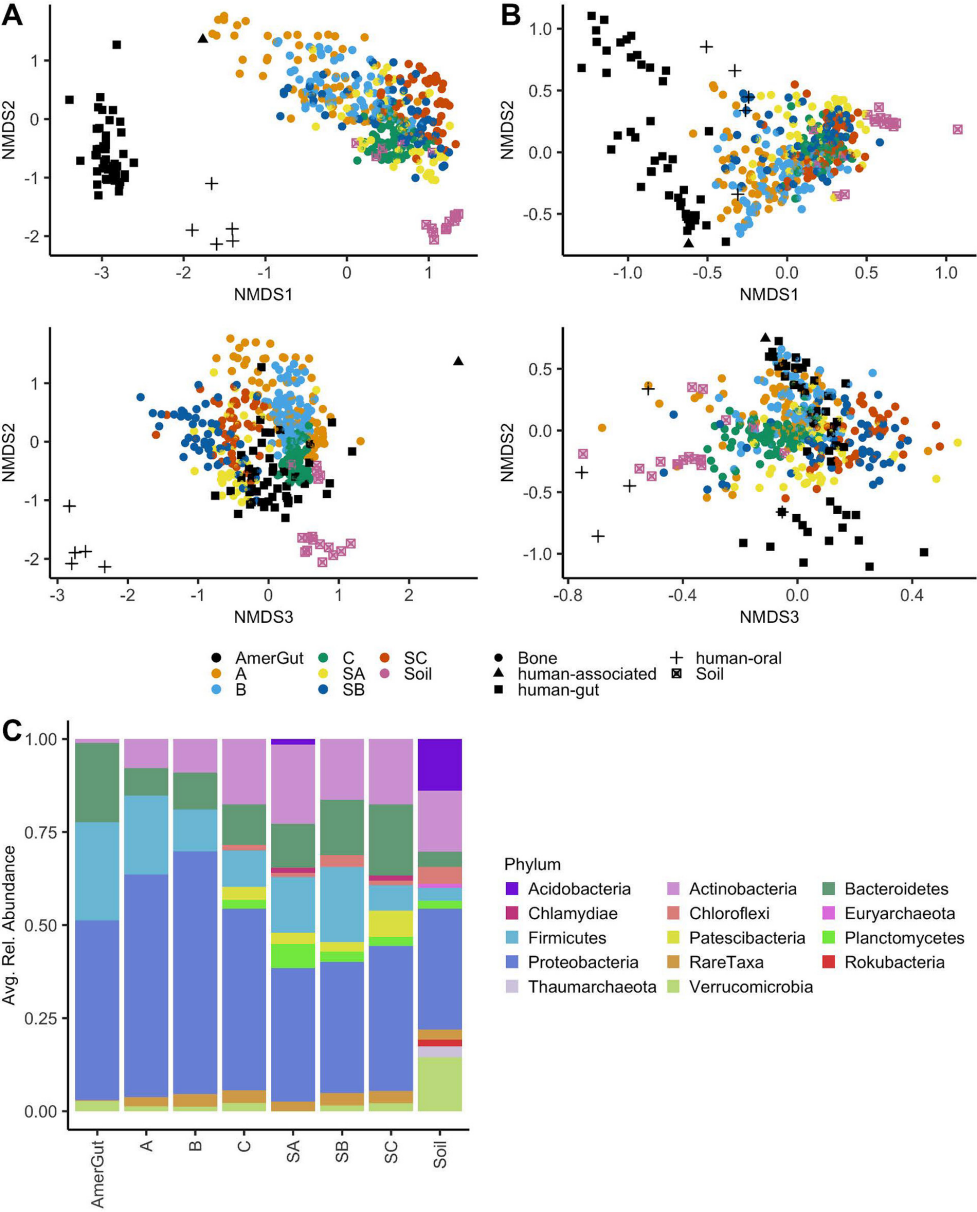
Nonmetric multidimensional scaling (NMDS) visualizing community composition differences in human bone, human-associated samples, and soils. Individuals included those from the grave (A, B, C) and those from the surface (SA, SB, SC), as well as individuals associated with the American Gut Project (AmerGut). (A) NMDS on Bray-Curtis dissimilarities (Stress = 0.102, k = 3, *n* = 453, n-taxa = 1,790); ASVs were merged at the genus level (B) NMDS on weighted UniFrac distances (Stress = 0.105, k = 3, *n* = 453, n-taxa = 11,263). Ordination applied to the ASV level. (C) Mean relative abundances (using rarefied data) averaged by individual (A, B, C, SA, SB, SC) or type (AmerGut and Soil). Only gut samples were included in AmerGut, and taxa with less than 1% relative abundance were denoted as “rare taxa.”

**FIG 2:**
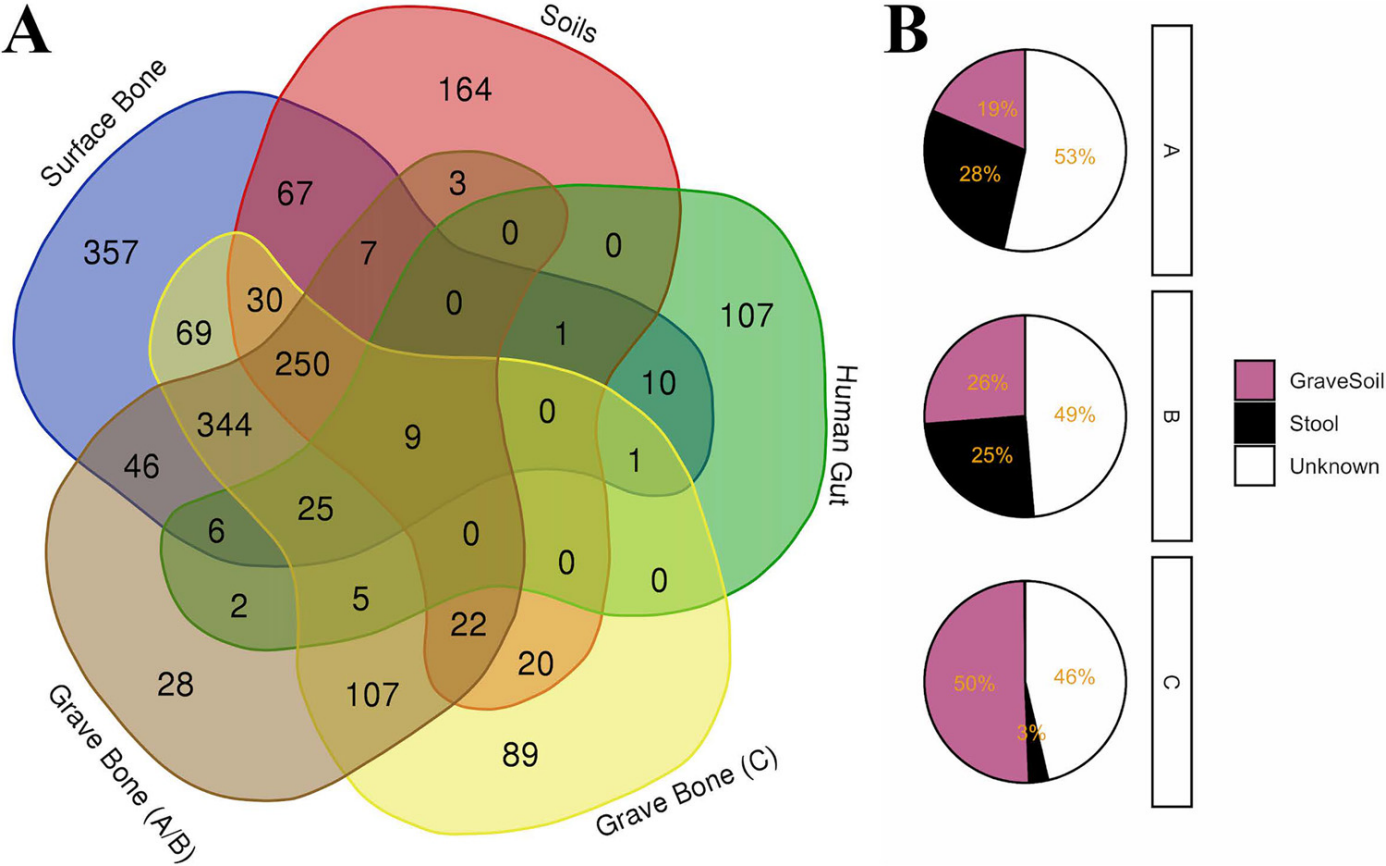
(A)Venn diagram of shared genera across groups (surface bone, buried bone, gut-associated samples, and soil). Buried individuals (A, B, and C) were split into two groups based on Bray Curtis or UniFrac distances and NMDS ordinations. (B) SourceTracker2 source proportions for buried bone samples averaged by individual; taxa used for sourcetracker2 were collapsed at the genus level (*n* genera = 1,790).

Bacterial and archaeal communities were significantly different by project (AGP, buried, and surface) and sample type (soil, human gut, human oral, and bone) based on PERMANOVA on dissimilarity/distance metrics (i.e., Bray-Curtis and weighted UniFrac) (Table S5). Bone communities, including bones from surface and buried contexts, were more similar to each other than they were to soil or human-associated communities (Fig. 1). When considering phylogenetic distance using weighted UniFrac, communities from buried individuals A and B, and especially A, showed greater distance from soil samples and increased similarity with human-associated communities from living donors. This pattern was reaffirmed at the phylum level compositionally (Fig. 1C). A Venn diagram was used to track the number of genera shared between the human gut, surface bone, buried bone, and soil. Excluding taxa shared between soil and human gut samples, a total of 49 taxa (2.74% of genera found in bone) were shared between bone and human gut samples, while 399 genera (22.3% of genera found in bone) were shared between bone and soil, leaving the origin of 1,040 genera (58.1% of genera found in bone) from decaying human bone unknown (Fig. 2). SourceTracker2 (21) was also applied, using grave soils (excluding 70 cm and 40 cm samples), off-grave soils, and human gut samples as sources and bone samples (buried) as sinks. The mean source contribution of buried bones varied by individual (Fig. 2B). The greatest mean source contribution across all buried bones was unknown (49.4%); grave soil was the second greatest source contributor with a mean source contribution of 32%, followed by stool (mean = 18.5%). Control soil samples contributed less than 1%. Control samples included in SourceTracker2 analyses were collected 0.5 m from the grave. Excluding a single sample at a depth of 30 cm, control samples were collected at a depth of 0 cm.

10.1128/msystems.00041-22.5 TABLE S5 PERMANOVAs (999 permutations), assessing between-sample differences (beta-diversity) using two metrics (Bray-Curtis dissimilarities and weighted UniFrac phylogenetic distances). Download Table S5, DOCX file, 0.01 MB. Copyright © 2022 Emmons et al. 2022 Emmons et al. https://creativecommons.org/licenses/by/4.0/ This content is distributed under the terms of the Creative Commons Attribution 4.0 International license.

Among bone communities, beta diversity was affected by the bone depositional environment (buried versus surface) and differences between individuals (A, B, C, SA, SB, SC) (Table S5). Individuals that decomposed on the ground surface clustered closely with buried individual C, the shallowest individual in the multi-individual grave (Fig. 1). When assessing samples from the grave project in isolation, bacterial and archaeal communities demonstrated significant differences by individual (A, B, and C) and anatomical region (skull, upper torso, arm, hand, lower torso, leg, and foot) (Table S5). This was, in part, influenced by differences in group variance. All factorial variables tested, with the exclusion of project (i.e., American gut, buried, or surface), showed significant differences in group variance, following tests for homogeneity of multivariate dispersion on Bray-Curtis dissimilarities (*P < *0.001). Samples from individual C showed less dispersion than those from A or B (Fig. 1).

**FIG 3:**
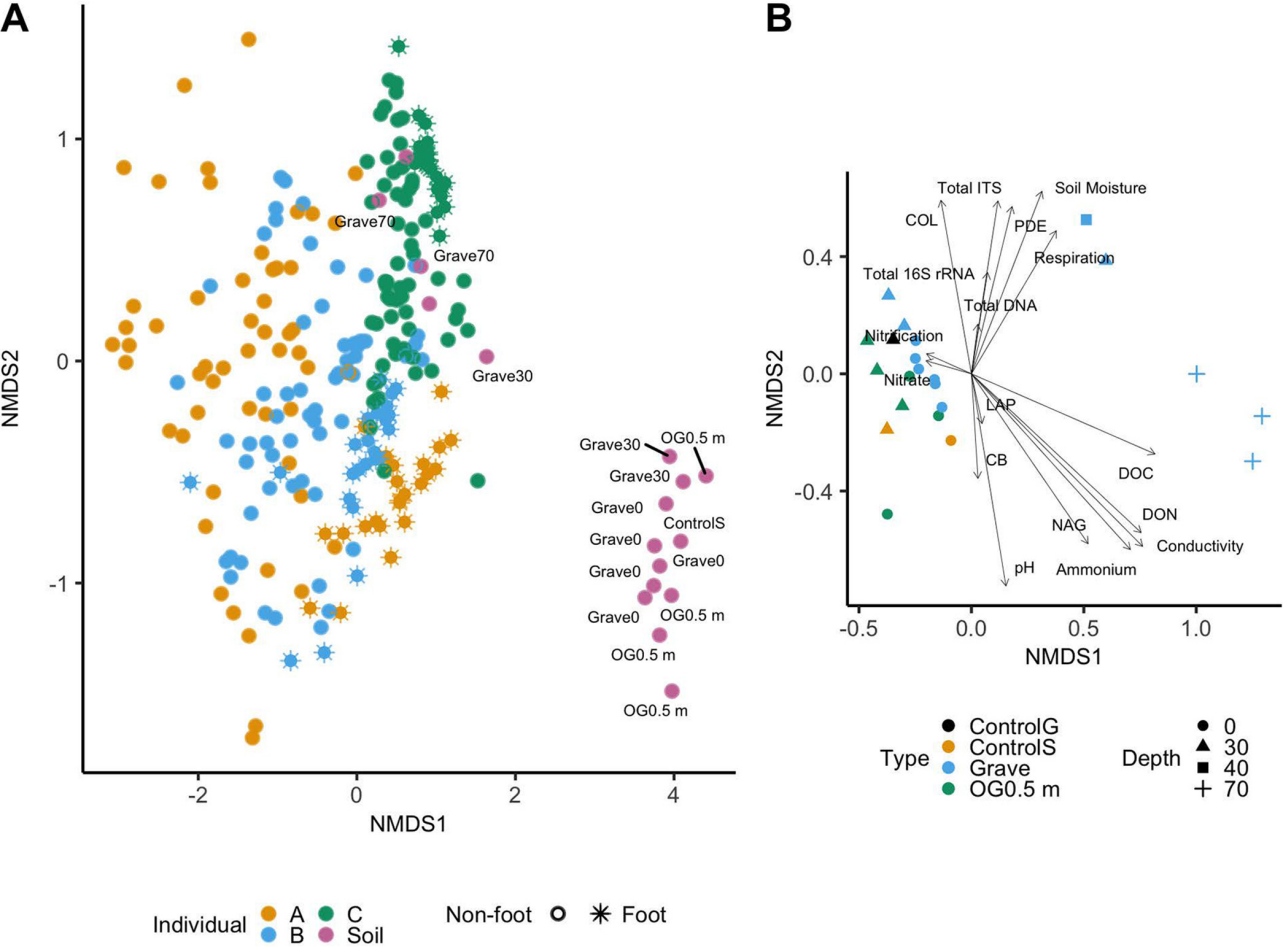
(A) Non-metric multidimensional scaling using Bray-Curtis dissimilarities of buried human bone samples and associated soils (stress = 0.128, k = 2, *n* = 259, n-taxa = 6,451). Soil samples are labeled by location (on-grave [Grave] or off-grave [OG 0.5 m]), depth within the grave (Grave 0 cm, 30 cm, 40 cm, and 70 cm), and type, including controls (ControlS). Asterisks indicate bone samples collected from the feet of buried individuals, and circles represent all other sample types. (B) NMDS of soil communities on weighted UniFrac distances with combined geochemistry using the envfit function from the package vegan (stress = 0.046, k = 2, *n* = 22, n-taxa = 3,090). Soil samples were rarefied to an even depth of 7,000 reads to include more soil samples in the analysis; taxa were assessed at the level of amplified sequence variants (ASVs). Colors refer to soil sample type (control grave samples [ControlG], control soil samples [ControlS], grave soil samples [Grave], and off grave soil samples at 0.5 m [OG0.5 m]). Shapes indicate depth.

Bones from individuals who decomposed on the surface and those at the shallowest grave depths showed high similarity with soil samples (Fig. 1 and 3). Though soil communities formed a distinct cluster, five soil samples (3 samples from a depth of 70 cm, 1 from a depth of 30 cm, and the mixed organic material collected at 40 cm) were closely associated with bone samples (Fig. 1A and B, and 3A). An NMDS of geochemistry and microbial community structure showed a significantly increased potential N-acetyl-glucosaminidase activity, dissolved organic carbon (DOC), and dissolved organic nitrogen (DON) at 70 cm and increased potential phosphodiesterase activity in the mixed organic material from 40 cm (Fig. 3B). In addition, though microbial communities were significantly different by anatomical region, a significant interaction between anatomical region and individual suggests that this was conflated by individual differences (Table S5). When visualized using NMDS on Bray-Curtis dissimilarities, foot bones were the only samples that demonstrated clear clustering across all three buried individuals (Fig. 3; Fig. S4).

10.1128/msystems.00041-22.9 FIG S4 NMDS of Bray-Curtis dissimilarities of subsurface bones by anatomical region. (A) NMDS ordination of individual A (Stress = 0.161, k = 2, *n* = 78). (B) NMDS ordination of individual B (Stress = 0.154, k = 2, *n* = 82). (C) NMDS ordination of individual C (Stress = 0.175, k = 2, *n* = 82). Ellipses represent 95% confidence intervals, as calculated using ggplot’s stat_ellipses() function. Download FIG S4, TIF file, 6.3 MB. Copyright © 2022 Emmons et al. 2022 Emmons et al. https://creativecommons.org/licenses/by/4.0/ This content is distributed under the terms of the Creative Commons Attribution 4.0 International license.

### Beta diversity: sample sites, sequencing runs, sampling date.

**FIG 4:**
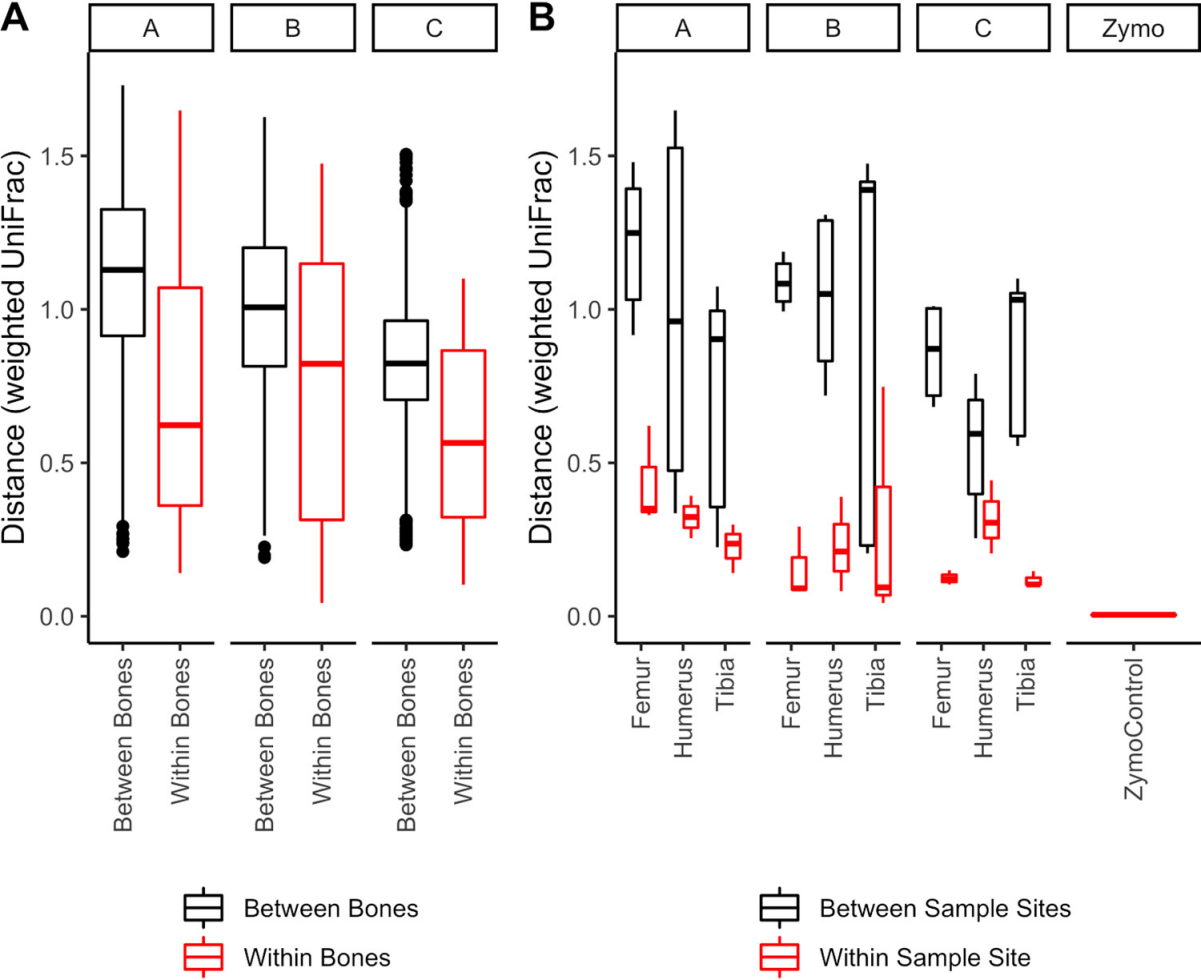
Weighted UniFrac distances demonstrating differences between bones, sample sites on the same bone, and samples collected from the same sample site. (A) Distances between bones and distances within bones (i.e., comparing multiple sample sites) within the same individual. (B) Only samples from the humeri, tibiae, and femora are shown to emphasize differences between technical replicates (replicate DNA extractions from a single bone powder sample). A Zymo DNA mock community control was included in each sequencing plate, and distances between Zymo replicates across plates are also shown here.

Weighted UniFrac was used to assess differences between samples from the same site (i.e., technical variants) and samples from different sites on the same bone. Replicates from a single site on the humerus, femur, and tibia were similar, as exemplified by small weighted UniFrac distances between samples, especially compared with distances between different sample sites on the same bone (Fig. 4B). The distal femur of A and the tibial midshaft of B were exceptions, with weighted UniFrac distances of 0.62 and 0.74, respectively. While samples from the distal femur of A were not homogenous at the time of sampling, the variation in the tibial midshaft of B remains unexplained.

Microbial communities from different sites on the same bone (e.g., midshaft and epiphysis) showed greater differences than those from the same site (Fig. 4A and B). Though not measured in this study, physical distance and bone microstructure appear to relate to microbial community differences between sample sites on the same bone. Larger bones with greater distances between sample sites (e.g., femur, humerus, tibia, radius, ulna, rib 7, clavicle) showed greater microbial community dissimilarity (data not shown). Small bones with increased weighted UniFrac distances between sample sites included the patella of B, navicular of A, talus of A, and MC4 of A.

Positive control DNA standards of known microbial community composition from Zymo Research were included in each sequencing run. Five of the eight bacterial genera were detected, and filtering successfully removed the fungal sequences. Community structure of the Zymo Research control was consistent across runs (weighted UniFrac distances: mean = 0.0044, min = 0.0029, max = 0.0057) (Fig. 4B).

To assess potential batch effects due to a lack of randomization, NMDS ordinations of Bray-Curtis distances were assessed by sequencing run and visualized by individual and skeletal sample collection date. The effect of sample type and individual are apparent regardless of sequencing run or sample collection date (data not shown).

### Similarity percentages (SIMPER) and differential abundance testing (CORNCOB).

Fifteen amplicon sequence variants (ASVs) identified by SIMPER analyses were significantly different between grave individuals (*P* < 0.01). Significant ASVs contributing to Bray-Curtis dissimilarities between grave individuals at the family level included Streptomycetaceae, Ruminococcaceae, Enterobacteriaceae, Burkholderiaceae, Rhizobiaceae, Pseudomonadaceae, Clostridiaceae I, Aquaspirillaceae, Rhodanobacteraceae, and Rhodocyclaceae. ASVs differentiating A and B from C were from the genera *Streptomyces*, *Caproiciproducens*, unclassified Burk-holderiaceae, Pseudomonas, unclassified Clostridiaceae, *Microvirgula*, unclassified Enterobacteraceae, *Rhodanobacter*, and *Ochrobactrum*. These ASVs were further tested for differential abundance using CORNCOB. Twelve of the 15 significant ASVs from SIMPER were significant following differential abundance testing (Fig. S5). ASVs from the genera Pseudomonas (2 ASVs), unclassified Enterobacteriaceae, *Azospira*, unclassified Burkholderiaceae (1 of 2 ASVs), *Microvirgula*, *Caproiciproducens*, and unclassified Clostridiaceae (2 ASVs) were associated with positive coefficients and expected to have greater relative abundances for individuals A and B compared with C. ASVs from the genera *Rhodanobacter*, unclassified Burkholderiaceae, and *Streptomyces* resulted in negative coefficients for A and B, indicating lower relative abundance in A and B compared with C.

10.1128/msystems.00041-22.10 FIG S5 Combined SIMPER and CORNCOB. Significant ASVs resulting from SIMPER were evaluated with CORNCOB. The dashed line (baseline) represents individual C. Differential abundance was tested across individuals while controlling for dispersion by individual. Download FIG S5, TIF file, 8.1 MB. Copyright © 2022 Emmons et al. 2022 Emmons et al. https://creativecommons.org/licenses/by/4.0/ This content is distributed under the terms of the Creative Commons Attribution 4.0 International license.

### Metabolomics.

**FIG 5:**
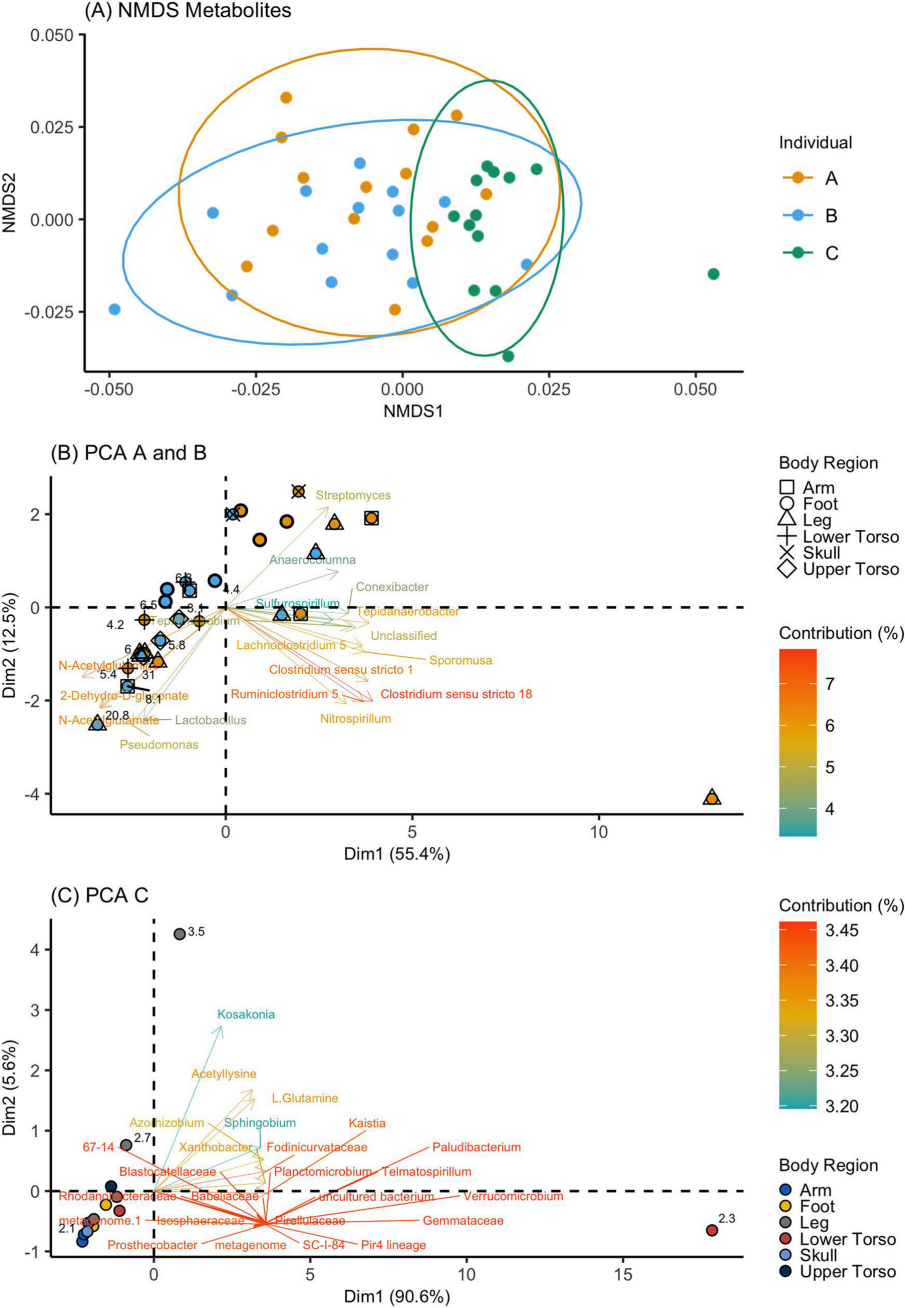
Metabolites and microbial communities. (A) NMDS ordination of Bray-Curtis distances calculated for known metabolites (stress = 0.19, k = 2). (B) sCCA to PCA of microbes and metabolites associated with individuals A and B. Samples are labeled with the human degradation index if greater than or equal to 3. (C) sCCA to PCA of microbes and metabolites associated with individual C. Samples are labeled with the human degradation index if greater than or equal to 2.

Known metabolic profiles, generated using Bray-Curtis distances and nonmetric multidimensional scaling, resembled taxonomic profiles (Fig. 5A). Using a PERMANOVA test, metabolite profiles demonstrated significant differences by individual (*F *= 4.58, R^2^ = 0.17, *P = *0.002, df = 2) and anatomical region (*F *= 2.17, R^2^ = 0.20, *P = *0.007, df = 5). Sparse canonical correlation analysis (sCCA) resulted in a correlation of 0.95 between the two matrices (metabolites and taxa) for individual C, with 30 features selected as capturing the most covariance. These features included 28 genera and 2 metabolites (acetyllysine and glutamine) (Fig. 5C). For individuals A and B, which were run independently from C due to significant differences in metabolite profiles, sCCA resulted in a correlation of 0.87, with 15 genera and 3 metabolites (N-Acetylglutamine, N-Acetylglutamate, and 2-Dehydro-d-gluconate) capturing the most covariance (Fig. 5B). These metabolites demonstrate a relationship with high human DNA degradation indices and correlate with relative abundances of Pseudomonas and Lactobacillus (Fig. 5B).

## DISCUSSION

### Microbial community differences between surface and subsurface bones.

Surface and buried bones showed differences in bacterial and archaeal community composition, especially in Planctomycetes, Chloroflexi, Chlamydiae, and Deinococcus-Thermus, which were more prevalent in surface remains. Chlamydiae, in surface remains, was dominated by the order Chlamydiales, with major representatives from Neochlamydia and Candidatus Protochlamydia. The phylum Chlamydiae includes obligate intracellular bacteria associated with a diverse range of hosts including arthropods, mammals (including humans), and amoeba (22). The two dominant genera sequenced from surface bone are likely amoebic endosymbionts, potentially from *Acanthamoeba* spp. or *Hartmannella* spp., which include free-living, bacterivorous amoeba found in diverse habitats (23, 24). Thermomicrobiales, mainly composed of an unclassified JG30-Kf-CM45, and Caldilineales were the dominant members of the phylum Chloroflexi in surface remains. The presence of Deinococcus-Thermus and Chloroflexi in surface bones, both of which include thermophilic members, potentially reflects extreme changes in the surface depositional environment, including temperature, moisture, and UV radiation.

Though microbial communities from surface and buried environments were significantly different in composition, microbial communities from bone appear to be a coalescence of human-associated gut communities and soil microbial communities, indicating a shared community of mixed origin. Surface bone microbial communities shared similarities with soil samples in terms of community composition and abundance, while buried communities demonstrated more proximate associations with gut samples, likely influenced by their decomposition state and oxygen availability in the grave. Excluding individual C, this was also true of alpha diversity analyses. Individuals A and B had significantly lower mean human DNA concentrations than C (25). While these patterns in microbial diversity could indicate that prolonged exposure to enteric bacteria increases the likelihood of skeletal DNA degradation (18–20), they are more likely related to differences in the physical and geochemical environment of A and B compared with C. While the grave was located in the unsaturated zone above the water table, many of the bones from A and B were submerged at the time of disinterment due to a perched water table that had formed within the grave, resulting in anoxic conditions and differences in geochemistry (26). These skeletal elements likely experienced fluctuations in site hydrology, which would impact both the microbial communities present and human DNA concentration and stability.

In alignment with surface observations, Damann et al. (27) observed shifts in surface exposed bone-associated communities with increasing postmortem interval, reflecting a convergence with soil communities over time. Similarly, Metcalf et al. (28) found that a minimum of 40% of microbial (archaeal and bacterial) decomposers could be linked to the soil environment and were found at low abundances prior to decomposition. Many bone colonizers were not observed in soil or gut samples in this study (Fig. 2). Though microbial populations could have multiple additional origins not captured by our analyses (water, skin, hair, etc.), more parsimonious explanations may include differences in initial sequencing depth and inadequate controls. For unknown reasons, soil samples had a much lower read distribution (∼30,000 reads) than bone samples. Though this was controlled in some capacity by rarefying ASV data to even sequencing depths, population migrations would be better identified with deeper sequencing of source samples. Additionally, soil samples below a depth of 30 cm (*n* = 5) were removed from Venn diagram and SourceTracker2 analyses because these samples were heavily influenced by decomposition by-products. Therefore, these analyses lacked a large subset of potentially anaerobic soil microbes that were in contact with decomposing remains. However, beta diversity ordinations indicate that soils influenced by decomposition cluster within bone samples, which may suggest that the decomposer community itself, is a potential source that influences postmortem bone communities. Alternatively, the postmortem interval, which was approximately 2 years for surface remains and 4.5 years for buried remains at the time of bone sampling, was great enough to allow a significant divergence from source populations. This may also have been amplified by the transfer of remains from the natural/outdoor environment to the laboratory environment for several months prior to sampling.

Microbial communities from individual C deviated from A and B, and demonstrated greater similarity with surface individuals. Potential factors responsible for this divergence include soil depth, oxygen availability, soil composition, pH, moisture, arthropod activity, and temperature. Individual C, recovered closest to the surface, was first exposed during excavation at a depth of 27 cm. Diurnal and seasonal temperature fluctuations as well as necrophagous insect activity have been observed in burials at depths less than 30.5 cm (16). In addition, changes in soil geochemistry within the grave indicate a shift in oxygen availability below 40 cm, coinciding with a change in community functionality, which was reflected in soil enzymatic potentials. Increases in N-acetyl-β-glucosaminidase and decreases in phosphodiesterase and leucine aminopeptidase at the grave base point to a more oligotrophic community (26).

Microbial communities from individuals A and B showed greater intra-individual dispersion, potentially as an effect of stacking the bodies within the grave, which likely promoted skin and tissue preservation on the torsos of A and B. Decomposition materials pooled to the grave base, primarily as a result of gravity. Soil encompassing individuals was disturbed during interment, decreasing soil compaction, and thereby promoting increased drainage to the grave base, creating a water bucket and sponge-like effect (29, 30). This was reflected in soil microbial communities at the grave base and further supported from a sample of mixed soil and organic material collected from within the rib cage of individual C. Microbial communities from the deep soil samples and mixed organic material in the rib cage deviated from the communities of other grave soil samples and were more similar in community composition and abundance to bone samples.

### Identifying bone colonizers and putative degraders.

We previously speculated about the potential impact of groundwater and its associated effects on differences in DNA survival between individuals (25). Though the grave base was saturated at the time of disinterment, decreased human DNA concentrations are indicative of fluctuating water levels within the grave (25). Seasonally water-logged sites exhibit poor skeletal preservation (31), which is expected given the mechanisms of bone diagenesis and the impact of water (3, 6).

Though we would have liked to have identified microbial taxa associated with high and low levels of human DNA preservation, including human DNA concentration and human DNA degradation index, individual differences and a lack of biological replicates with depth made achieving this goal extremely difficult. Individuals A and B had some of the lowest human DNA concentrations and highest human DNA degradation indices (25). They also had relatively higher abundances of genera Pseudomonas, unclassified Enterobacteriaceae, *Azospira*, unclassified Burkholderiaceae, *Microvirgula*, *Caproiciproducens*, and unclassified Clostridiaceae. Two potential mechanisms through which microbes can invade and degrade bone include the production of collagen specific proteases known as collagenases and organic acids (9, 32), which would release bound DNA from bioapatite and/or contribute to the degradation of DNA (8). Pseudomonas, in particular, may play an important role in skeletal degradation given its known ability to solubilize inorganic and organic phosphate (33–35). Phosphorus is an important nutrient, critical to multiple major biological processes (35). Bioapatite, the inorganic material in bone, which is closely related to hydroxyapatite, is a good source of inorganic phosphorus (36). Using combined 16S rRNA sequencing and untargeted metabolomics, we discovered a positive correlation between relative abundances of Pseudomonas, *Lactobacillus*, and three metabolites, one of which included 2-dehydro-d-gluconate (a.k.a., 2-ketogluconate), as well as a potential relationship with human DNA degradation indices. The human DNA degradation index is a measure of DNA degradation; it is a ratio of the quantity of human DNA resulting from a small 80 bp amplicon compared with a large 214 bp amplicon (25) and is included here as a proxy for overall skeletal integrity. This result is significant because the general mechanism through which Pseudomonas spp. solubilize inorganic phosphate involves the production of gluconic and/or 2-ketogluconic acids by way of the extracellular direct oxidative metabolism of glucose (33). 2-ketogluconic acid has demonstrated efficient solubilization of inorganic phosphate from calcium phosphates including hydroxyapatite (37).

### Conclusions and limitations.

This research furthers our understanding of postmortem bone colonization by microbes and its potential effects on human skeletal DNA preservation. In this work, we demonstrated that the postmortem bone microbiome was distinct from other potential source environments including the human gut and soil. However, we also showed that the communities in bone were related to the geochemistry of the depositional environment, with the deeper remains more like human gut samples with overrepresentation of anerobic taxa. Without further biological replicates and alternative forms of evidence (e.g., histology, metatranscriptomics, proteomics, controlled laboratory experiments, etc.), no direct link could be made between microbial taxa and bone degradation or skeletal DNA preservation. However, evidence from our combined metabolomic and amplicon sequencing analysis suggested that Pseudomonas and phosphate solubilization play a role in skeletal degradation, which warrants further investigation.

There exists an intricate relationship between physical, geochemical, and biological processes in the burial environment, which together influence bone diagenesis and skeletal DNA preservation. These influences are difficult to disentangle using actualistic taphonomic research with limits on sample size and destructive testing. Buried individuals included three stacked individuals in a single grave. The rationale for stacked placement was to replicate a realistic mass grave scenario. However, due to the difficulty of obtaining a large number of willfully donated human remains for decomposition research, especially when sampling is destructive, we were unable to inter multiple sets of remains. This limits our ability to make definitive conclusions with the data at hand. Moreover, due to storage conditions, at room temperature prior to skeletal sampling, and a failure to adequately randomize samples, taxa blooms and batch effects are possible. Nevertheless, an evaluation of included community controls, skeletal sampling time points, and sequencing runs seems to suggest our observed patterns were not due to sample processing or sequencing artifacts. Lastly, using DNA as a target, we had no way to decipher primary bone colonizers and/or degraders from secondary or tertiary beneficiaries and other syntrophic bacteria or incidental taxa. Microbes important for early diagenetic processes could become embedded within the bone matrix during cycles of demineralization and recrystallization (38). Ultimately, more evidence with greater replication and randomization is necessary to disentangle the impact of the postmortem bone microbiome on skeletal DNA preservation. This research adds to the body of evidence on the postmortem bone microbiome. As this evidence continues to grow, we come one step closer to understanding the overall impact of the grave environment, including that of the microbes, on skeletal preservation.

## MATERIALS AND METHODS

### Sample description.

In 2013, three human decedents, two males and one female, were interred in a multi-individual grave at the Anthropology Research Facility (ARF) at the University of Tennessee, Knoxville (UTK). In 2017, 4 years following interment, the grave was excavated, and the remains were disinterred to examine inter- and intra-individual patterns of skeletal DNA degradation (25) (Table 1). The DNA results were also to be compared to results from a study examining individuals who decomposed on the ground surface at the ARF (13) (Table 1). Project subjects were individuals who donated their bodies after death to the Forensic Anthropology Center (FAC) Body Donation Program at UTK. Because no living human subjects were involved in this research, the Assurance Status of this Project was Exemption Status under section 101(b), paragraph 4, by the UTK Institutional Review Board. Individuals (A, B, and C) were stacked crisscross to replicate a realistic multi-individual grave scenario. Individual A was located at the grave base; B was in the middle, and C was the shallowest. Simultaneously, a second unit 4.5 m away was excavated and backfilled, without interring human remains, to serve as a control grave. The two graves were approximately 2 m × 2 m × 0.7 m and 2 m × 4 m × 0.7 m, respectively, and located in an area of the ARF not previously used for decomposition research.

**TABLE 1:** Demographics of skeletonized individuals and duration of decomposition time.

ID	Project	Sample type	Wt (kg)	Age (yrs)	Medical history	Sex	Residence	Exposure/burial duration	Approximate time since death at skeletal sampling
SA	Surface	Bone	104	50	Diabetes, alcoholic, substance abuser	Male	TN/AL	13 mo	2 yrs
SC	Surface	Bone	127	69	Diabetes, cardiac issues	Male	TN	16 mo	2 yrs
SB	Surface	Bone	80	47	High cholesterol, arthritis	Male	TN	23 mo	2 yrs
B	Grave	Bone	70	63	COPD	Male	VA	4 yrs	4.5 yrs
A	Grave	Bone	60	68	Cancer	Female	WA	4 yrs	4.5 yrs
C	Grave	Bone	52	63	Cancer, coronary artery dx	Male	GA/AL	4 yrs	4.5 yrs

### Soil samples.

Soil samples were collected during disinterment at four depths (0–5 cm, 30–35 cm, 70–75 cm, and 80–85 cm) within the perimeter of the grave and at two depths (0–5 cm, 30–35 cm) along three lateral transects extending away from the grave. An additional sample of mixed soil and organic material was collected from within the rib cage of individual C, at ∼40 cm depth. Control soil samples were collected at two depths (0–5 cm and 30–35 cm) from two locations. The first location was an undisturbed area at least 5 m from the grave and other sites of decomposition, while the second was from the control grave. Soil samples were sent to the UTK Department of Biosystems Engineering and Soil Sciences for soil biological and geochemical testing. Variables tested included pH, conductivity, extracellular enzyme potentials (leucine aminopeptidase, N-acetyl-glucosaminidase, collagenase, and phosphodiesterase), soil gravimetric moisture, microbial respiration, ammonium, nitrification potential, dissolved organic nitrogen and carbon, and nitrate. Soils were also tested for total fungal and bacterial gene abundances, human-associated Bacteroides gene abundances, and soil nematode abundance and composition. In-depth details on soil collection, analyses (including soil DNA extraction methodology), and results have been reported in Keenan et al. (26). DNA extracts from a subset of these samples were used for amplicon library sequencing (*n* = 27) (Table S1).

10.1128/msystems.00041-22.1 TABLE S1 Soil samples sent for sequencing. Download Table S1, DOCX file, 0.01 MB. Copyright © 2022 Emmons et al. 2022 Emmons et al. https://creativecommons.org/licenses/by/4.0/ This content is distributed under the terms of the Creative Commons Attribution 4.0 International license.

### Bone samples from burials.

Forty-nine skeletal elements from each of the 3 individuals, representing all skeletal element types, were sampled for human DNA, total DNA, and total bacterial and fungal gene abundances (n-elements = 147, n-samples = 247) (Table S2). Exceptions during sampling did occur causing some differences in sample size by element type. For example, the vertebral column of individual B was fused, so the cervical vertebrae could not be sampled. To examine intra-element variability, 19 of the 49 bones from each individual were sampled at 2 different sites on the bone (e.g., midshaft and proximal end), and 3 bones from each individual (humerus, femur, and tibia) were sampled at 3 sites on the bone. The sample sites from the humerus, femur, and tibia were also sampled twice at the same site on the bone to examine intrasite variability. Skeletal sampling took place over a 4–6-month period, beginning with individual A and ending with individual C; all bones were stored in cardboard boxes at room temperature prior to sampling.

10.1128/msystems.00041-22.2 TABLE S2 Buried skeletal samples by element. Download Table S2, DOCX file, 0.02 MB. Copyright © 2022 Emmons et al. 2022 Emmons et al. https://creativecommons.org/licenses/by/4.0/ This content is distributed under the terms of the Creative Commons Attribution 4.0 International license.

**FIG 6:**
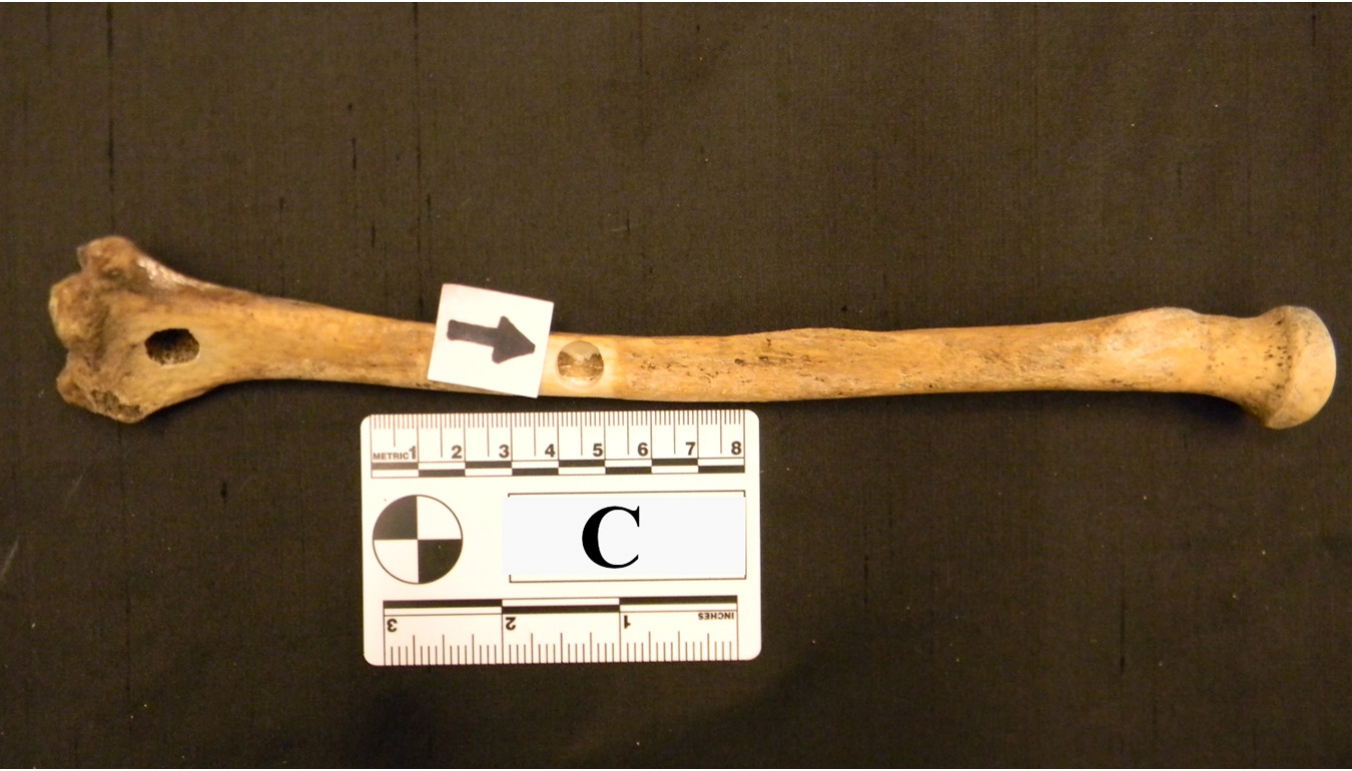
Left radius from individual C. The outer surface of the bone was mechanically removed using a Dremel rotary tool. The two sample sites are indicated by arrows.

At each sampling site the outer surface of bone was mechanically removed with a Dremel rotary tool and then chemically treated with 10% bleach and 70% ethanol prior to sampling. Bones were sampled using a handheld drill with a 9-mm masonry bit at low speed (Fig. 6). Samples were sent to Bode Technology, Lorton, Virginia, for DNA extraction and human DNA testing (e.g., Quantifiler Trio DNA Quantification, GlobalFiler PCR Amplification). Extracts were returned to UTK for total DNA and total bacterial and fungal gene quantification. Methods including skeletal DNA extraction, human DNA testing, and microbial gene quantification are reported in Emmons et al. (25). All skeletal DNA extracts used in Emmons et al. (25) were also used in the current study to assess bacterial and archaeal community structure (*n* = 247).

### Comparative samples.

Two additional data sets were included for comparative purposes. First, DNA sequences from the buried remains were compared directly to sequences from a previous data set composed of skeletal DNA extracts from three individuals (SA, SB, and SC) who had decomposed on the ground surface (*n* samples = 162, 54 samples per individual). These samples were sequenced on an Illumina MiSeq platform targeting the V3-V4 region of the 16S rRNA gene using 300 PE chemistry and have previously been characterized by Emmons et al. (13) and additionally reported on by Mundorff and Davoren (39). Second, sequence data associated with samples from human feces (*n* = 53), sebum (*n* = 2), saliva (*n* = 6), and hair (*n* = 1) and associated metadata were accessed from the American Gut Project (AGP) (Qiita Accession ID 10317), a crowd-sourced project including thousands of samples from multiple body sites, with an emphasis on feces (40). The additional data set samples were selected based on donor age (50–70 years) and state of residence (TN, GA, AL, WA, and VA) to mirror the demographic information from the skeletonized individuals (Table 1; Table S3). AGP samples were processed and sequenced according to Earth Microbiome Project (EMP) protocols using 150 PE chemistry on a MiSeq Illumina Platform (Primers 515F–806R) (41).

10.1128/msystems.00041-22.3 TABLE S3 Samples from the American Gut Project (Qiita 10317). Download Table S3, DOCX file, 0.02 MB. Copyright © 2022 Emmons et al. 2022 Emmons et al. https://creativecommons.org/licenses/by/4.0/ This content is distributed under the terms of the Creative Commons Attribution 4.0 International license.

### Next generation sequencing analysis.

DNA extracts associated with the grave, including soils and skeletal samples, were sent for next generation sequencing (NGS) at the Center for Environmental Biotechnology (CEB), UTK. Library preparation was performed by the CEB using the Nextera DNA Library Prep Kit according to manufacturer's instructions. Primers used were consistent with EMP protocols (515F–806R), targeting the V4 region of the 16S rRNA gene (41). Samples were sequenced on an Illumina MiSeq System using 250 PE chemistry. Two PCR blanks and one positive control (ZymoBIOMICS Microbial Community DNA Standard, Zymo Research) were included in each run. Extraction blanks were not included in NGS runs; all extraction blanks were previously tested for human DNA quantities (<0.001 ng μL^−1^), and a subset (12 of 25) was tested for total DNA using previously described methods (13, 25) (mean total DNA = 0.0006 ng μL^−1^, min = 0.0003 ng μL^−1^, max = 0.001 ng μL^−1^).

Sequence reads were processed using the QIIME 2 next-generation microbiome bioinformatics platform (v. qiime2-2018.11.0 and qiime2-2019.10; QIIME2 Development Team, 2018) (42). Read quality was assessed using QIIME2 demux; reads were quality filtered, denoised, chimeras removed, and demultiplexed using DADA2 (43). Primers from grave samples, soil, and bone were trimmed using the –trim-left function of the DADA2 plugin. Because bone samples from the comparative surface data set targeted V3-V4, sequences were trimmed, including primer removal, using EMP primers (515F–806R) targeting V4 using the QIIME2 cutadapt plugin prior to DADA2 (44). Sequencing runs were denoised independently and merged. While most reads were trimmed to ∼253 bp, there was variation in read length (min = 144 bp, max = 425 bp, mean = 263 bp), which resulted in 35,756 unique features (*n* = 507). Read loss varied by data set following denoising. Grave samples experienced a total read loss of 13.1%. Similar to previously reported values, total reads from surface bones were reduced by 64.1% (13). AGP samples saw a reduction in total reads by 30.4%. The maximum number of sequencing reads amplified from negative PCR amplification blanks included in subsurface sequencing runs was 249, while the minimum was 21.

Features, or amplicon sequence variants (ASVs), were classified using a fitted classifier (classify-sklearn) using the SILVA rRNA database v132 (silva-132-99-515-806-nb-classifier.qza) (45). A rooted phylogenetic tree was generated using the q2-fragment-insertion plugin (46); SILVA v128 (sepp-refs-silva-128.qza) was used as the reference database and backbone of the tree. ASVs identified as mitochondria, chloroplasts, and eukaryotes were filtered from the feature table, as well as unidentified ASVs at the domain level. Feature and taxonomic data were exported to R (v. 3.5.0) (47) for statistical analyses and visualization using phyloseq (v. 1.20.0) (48). ASVs not observed across a minimum of 0.5% of samples (∼2) were also removed, resulting in 11,386 ASVs (see reference 49). Samples were rarefied to an even depth of 10,000 reads to account for uneven library sizes (50) prior to alpha and beta diversity measurements including ordination methods and visualizations based on ordinations. While this depth resulted in sample loss (30 samples removed) and a reduction in the number of ASVs (n-taxa remaining = 11,263), rarefaction curves indicate that a depth of 10,000 reads adequately captured species richness for the majority of samples (Fig. S1). Tooth samples, from the surface data set, and positive controls were removed prior to most analyses, resulting in a total sample size of 453. Bray-Curtis and weighted UniFrac were computed for beta diversity analyses using phyloseq and qiime2, respectively. Alpha diversity metrics (Inverse Simpson and observed richness) were computed using a subsampling approach, in which richness and diversity metrics were computed and averaged for a total of 100 iterations, each scaled to even depth. Due to inherent differences between data sets (AGP, surface bone, and grave) and variation in sequence length as a result of next generation sequencing analysis, ASVs were combined at the genus level when all data sets were merged, excluding UniFrac analyses, as the use of a SEPP tree can overcome differences related to read length (46).

### Microbial community analysis.

Data analyses were conducted in R (v. 3.5.0) (47). Kruskal-Wallis tests were used to assess statistical significance in alpha diversity metrics with false discovery rate corrected *P* values to account for multiple comparisons. Group differences in beta diversity metrics (weighted UniFrac and Bray-Curtis) were assessed visually using nonmetric multidimensional scaling (NMDS) and statistically using permutational multivariate analysis of variance tests (PERMANOVAs), with a total of 900 permutations (vegan v. 2.5-3) (51). Multiple PERMANOVAs were performed; groups tested included project type (AGP, surface bone, and grave), sample type (human-gut, human-oral, bone, soil), bone environment (surface and grave), individual (A, B, C, SA, SB, and SC), grave individual (A, B, and C), and anatomical region (skull, upper torso, arm, lower torso, leg, foot). Tests for homogeneity of multivariate dispersion were also applied to grouping variables; 999 permutations were used.

SourceTracker2 (21) and a Venn diagram were used to explore potential origins of microbial source populations found in human bone. ASVs were rarefied to 10,000 reads and combined at the genus level. Soil samples at 70 cm and 40 cm were not included in either analysis, as these samples were determined to be affected by decomposition upon visualization of weighted UniFrac and Bray-Curtis distances. The Venn diagram and associated output were created using http://bioinformatics.psb.ugent.be/cgi-bin/liste/Venn/calculate_venn.htpl. For Sourcetracker2, source populations included grave and control soils as well as gut samples from the AGP. Bone samples were sink samples. The command line functionality and Gibbs function were used with default parameters, excluding the –source_rarefaction_depth and –sink_rarefaction_depth, which were set to 0.

SIMPER and similarity percentages, followed by nonparametric Kruskal-Wallis tests with false discovery rate (FDR) corrected *P* values, were used to determine ASVs significantly contributing to Bray-Curtis dissimilarities between grave individuals (seq-scripts release v. 1.0) (52). ASVs were filtered using a prevalence threshold of 1% from a sample data set that included only bone samples. ASVs were considered significant with an alpha < 0.01. These ASVs were further assessed using Count Regression for Correlated Observations with the Beta-binomial (CORNCOB) (53). CORNCOB was used to test differential abundance of ASVs from SIMPER across individuals, while controlling for the effect of individual on dispersion.

### Untargeted metabolomics.

Ultra high performance liquid chromatography–high-resolution mass spectrometry (UHPLC-HRMS) was applied to a subset of skeletal samples (*n* = 41) collected from buried individuals. Sample selection was arbitrary, based on the ability to obtain an additional 0.2 g of bone powder from a single sampling site, to minimize further element destruction. A minimum of 13 samples were included per individual (A: *n* = 14; B: *n* = 14; C: *n* = 13; Table S1). Samples were immediately frozen using liquid nitrogen and stored at −80°C pending metabolite extractions. Metabolite extraction and mass spectrometric analysis were performed at the Biological and Small Molecule Mass Spectrometry Core (BSMMSC) at the University of Tennessee, Knoxville.

Bone powder samples (30–50 mg) were suspended in 1.3 mL of extraction solvent (40:40:20 HPLC grade methanol, acetonitrile, water with 0.1% formic acid), prechilled at 4°C. To assure extraction and instrumental reproducibility, four stable isotope labeled internal standards, orotate-^15^N2, vanillin-^13^C6, glutamate-d5, and alanine-d4, were spiked in with a concentration of 100 nM. Samples were vortexed and allowed to extract for 20 min at 4°C while being shaken on an Orbital Platform Shaker (Bellco, Vineland, NJ). Once the extraction was complete, samples were centrifuged (5 min, 16,100 rcf, 4°C) and the supernatant was collected and transferred to new Eppendorf tubes. An additional 200 μL of extraction solvent was added to the residual bone powder and re-extracted as previously described. The supernatants were combined and dried to completion under a stream of nitrogen (2–3 h). The resulting dried residue was resuspended in 300 μL of sterile HPLC grade water and transferred to 300 μL autosampler vials and immediately analyzed.

For UHPLC-HRMS analyses, a 10 μL aliquot was injected through a Synergi 2.5 micron reverse-phase Hydro-RP 100, 100 × 2.00 mm LC column (Phenomenex, Torrance, CA), kept at 25°C. The eluent was introduced into the mass spectrometer (MS) via an electrospray ionization (ESI) source conjoined to an Exactive Plus Orbitrap mass spectrometer (Thermo Scientific, Waltham, MA) through a 0.1 mm internal diameter fused silica capillary tube. The MS was run in full scan mode with negative ionization mode with a window from 80–1200 *m/z* (54). Samples were run with a spray voltage of 3 kV. The nitrogen sheath gas was set to a flow rate of 25 lb/in^2^ with a capillary temperature of 300°C. AGC (acquisition gain control) target was set to 3e6. The samples were analyzed with a resolution of 140,000. A scan window of 72 to 800 *m/z* was used from 0 to 9 min and of 110 to 1000 *m/z* from 9 to 25 min. A flow rate of 0.2 mL/min was maintained throughout the run. Chromatography ran for a total of 25 min with a solvent A consisting of 97:3 HPLC grade water:methanol, 10 mM tributylamine, and 15 mM acetic acid. Solvent B was HPLC grade methanol. The gradient was as follows: 0 to 5 min, 0% B; 5 to 13 min, 20% B; 13 to 15.5 min, 55% B; 15.5 to 19 min, 95% B; 19 to 25 min, 0% B. Duplicate injections were performed for each sample.

After UHPLC-HRMS analysis, raw files generated by Xcalibur were converted to the universal mzML format (55) via the open-source MSConvert software as part of the ProteoWizard package (56). Metabolomic Analysis and Visualization Engine (MAVEN) software from Princeton University (57, 58) was used to automatically correct the total ion chromatograms based on the retention times for each sample. Known (annotated) metabolites were manually selected based on mass accuracy (± 5 ppm mass tolerance) and retention times (≤ 2 min).

Sparce canonical correlation analysis (sCCA) paired with principal-component analysis (PCA) was used to uncover relationships between significantly correlated known metabolites and microbial genera using the R packages PMA (Penalized Multivariate Analysis, v. 1.1) and ade4 (Analysis of Ecological Data: Exploratory and Euclidean Methods in Environmental Sciences, v. 1.7.16), respectively (59, 60). sCCA (penaltyx/penaltyz = 0.15) was used as a data reduction tool; reduced data were then visualized and assessed using PCA (per reference 49). All metabolites were log transformed (base 10) prior to analyses. A pseudocount of one was included to eliminate problems with zeros in log transformation. The ASV table included in sCCA analyses was rarefied to 10,000 reads. Rare taxa were removed, and the table was collapsed at the genus level to further reduce the number of features assessed.

### Data availability.

Raw sequence data is available at NCBI Sequence Reach Archive, Accession PRJNA725545. Accessory files including R code, associated output, and metadata are available on GitHub (https://github.com/aemmons90/Subsurface_Bone_Microbe). Skeletal material used in this project has been accessioned in the William M. Bass Donated Skeletal Collection at the University of Tennessee, Knoxville.
