# Supplementary figures and images for: Postmortem Skeletal Microbial Community Composition and Function in Buried Human Remains
Source: mSystems. 2022 Mar 30;7(2):e00041-22. doi: 10.1128/msystems.00041-22 (PMC9040591; doi:10.1128/msystems.00041-22)

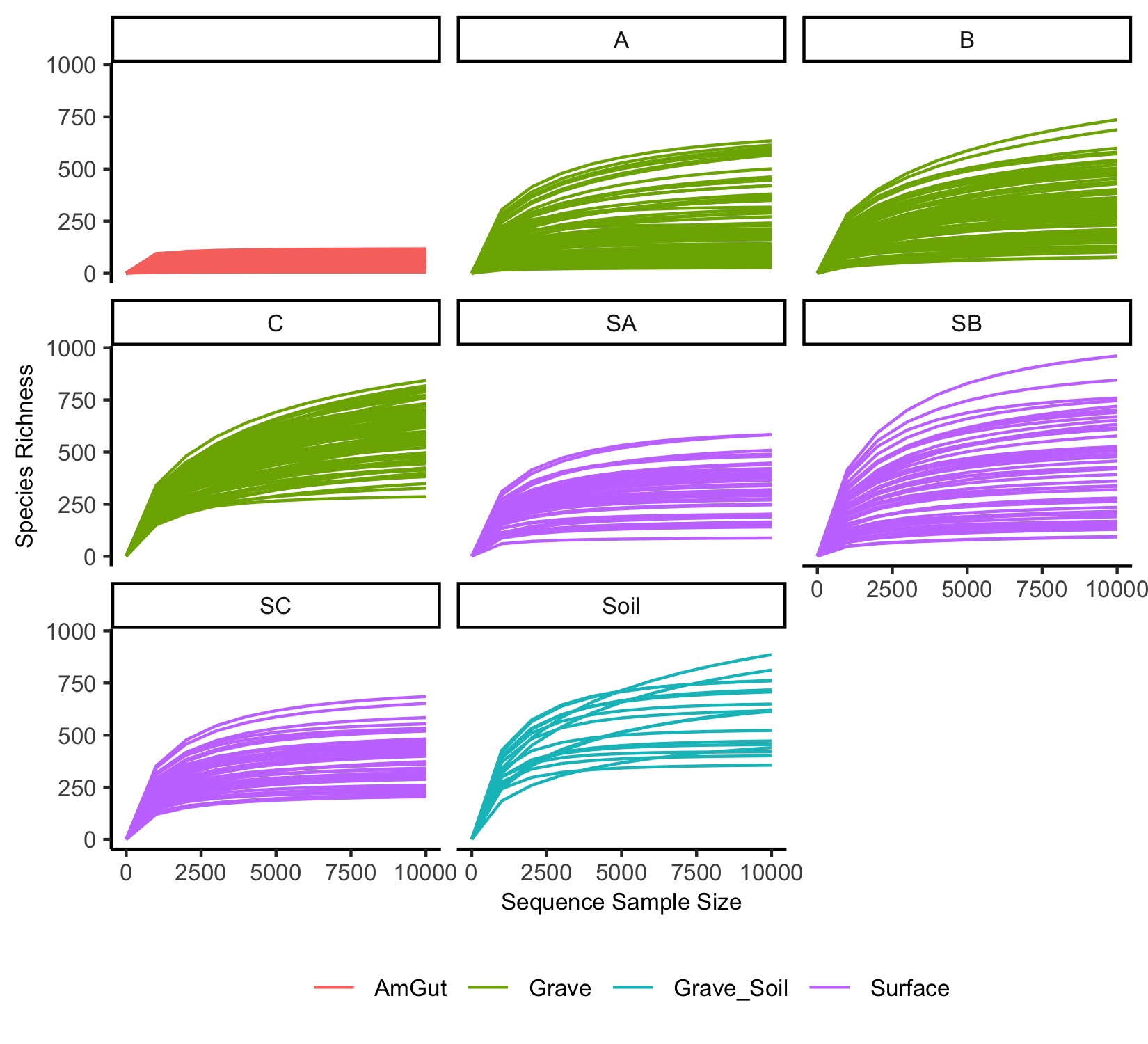

Supplement: FIG S1 [file msystems.00041-22-sf001.tif]

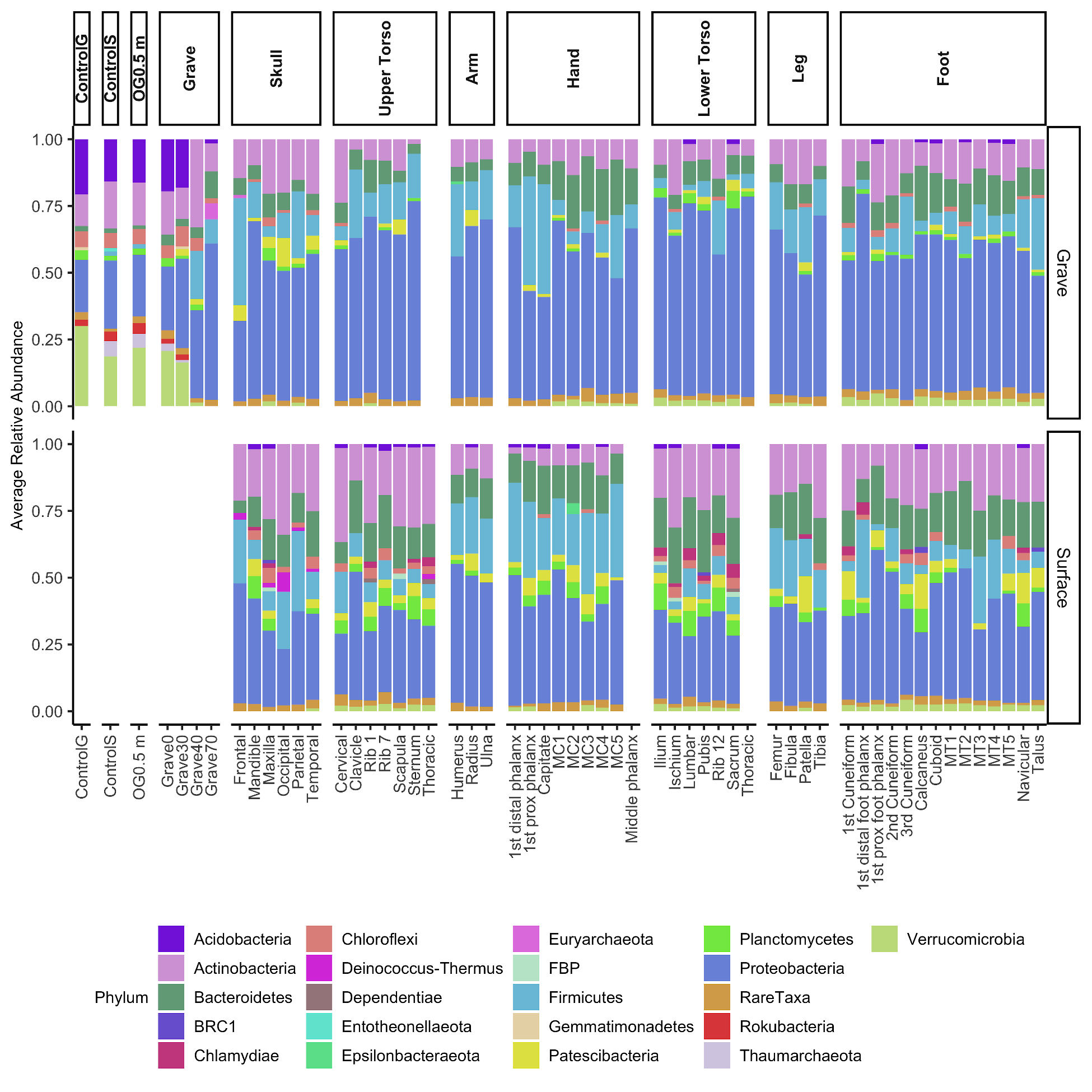

Supplement: FIG S2 [file msystems.00041-22-sf002.tif]

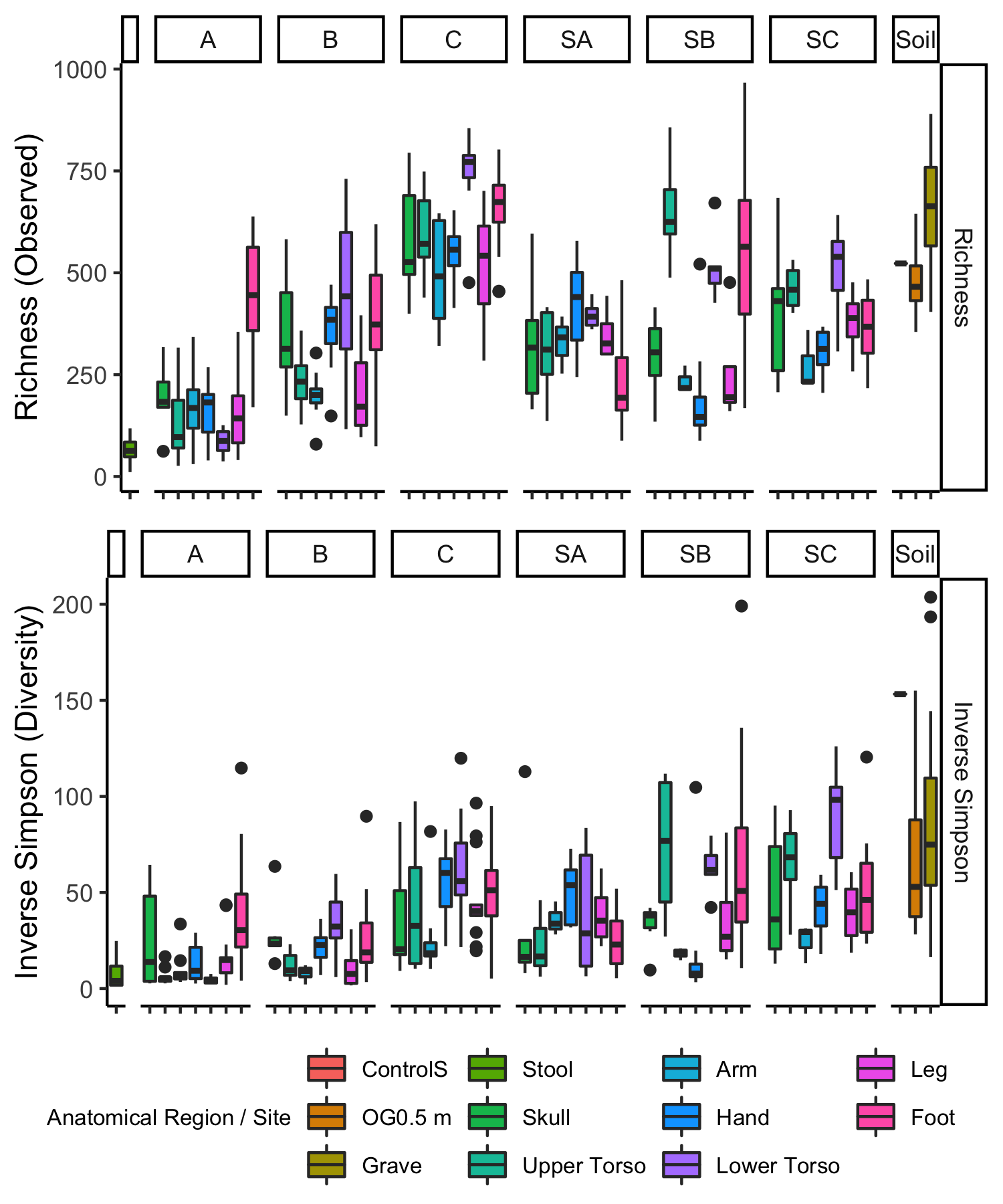

Supplement: FIG S3 [file msystems.00041-22-sf003.tif]

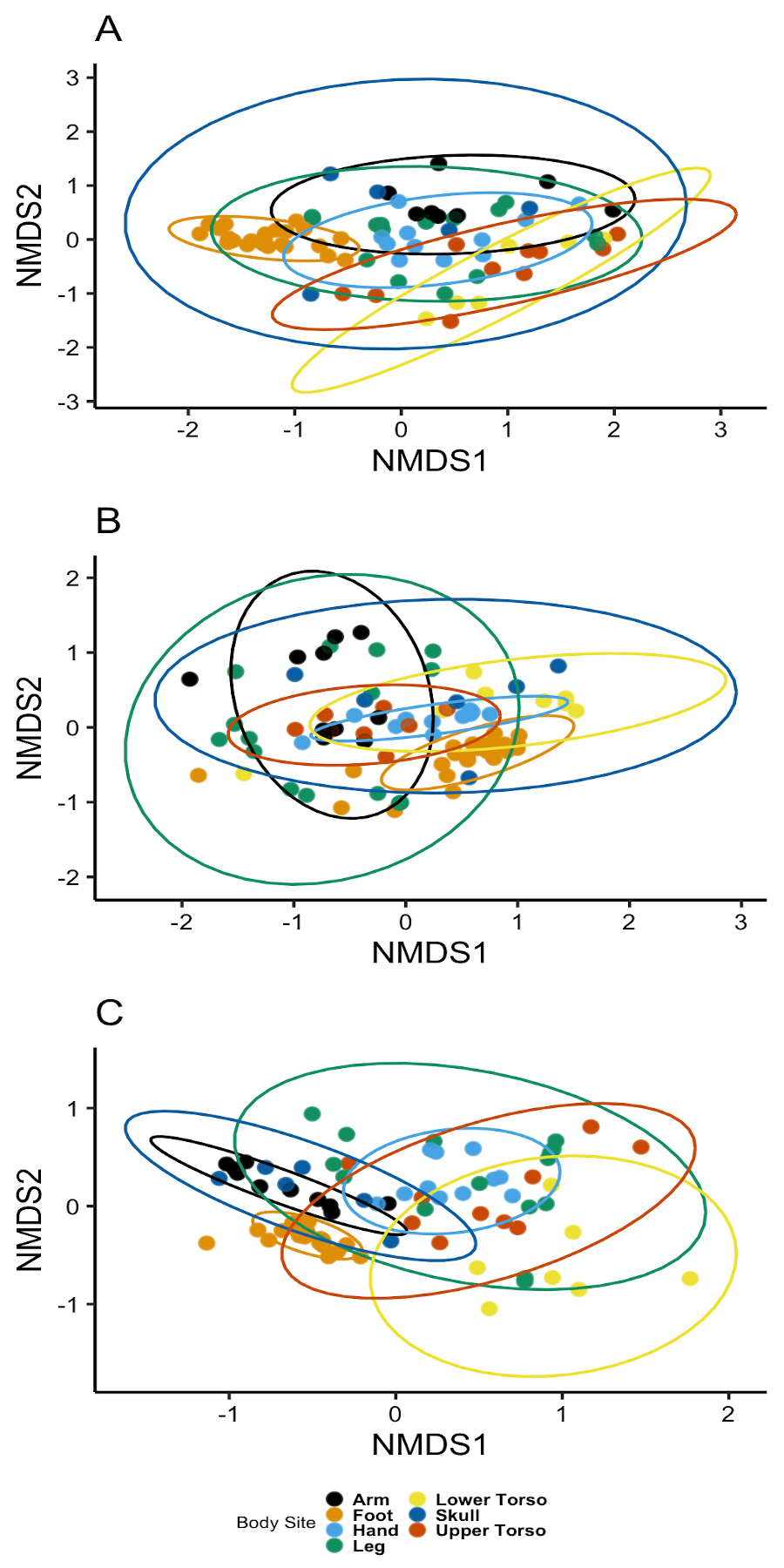

Supplement: FIG S4 [file msystems.00041-22-sf004.tif]

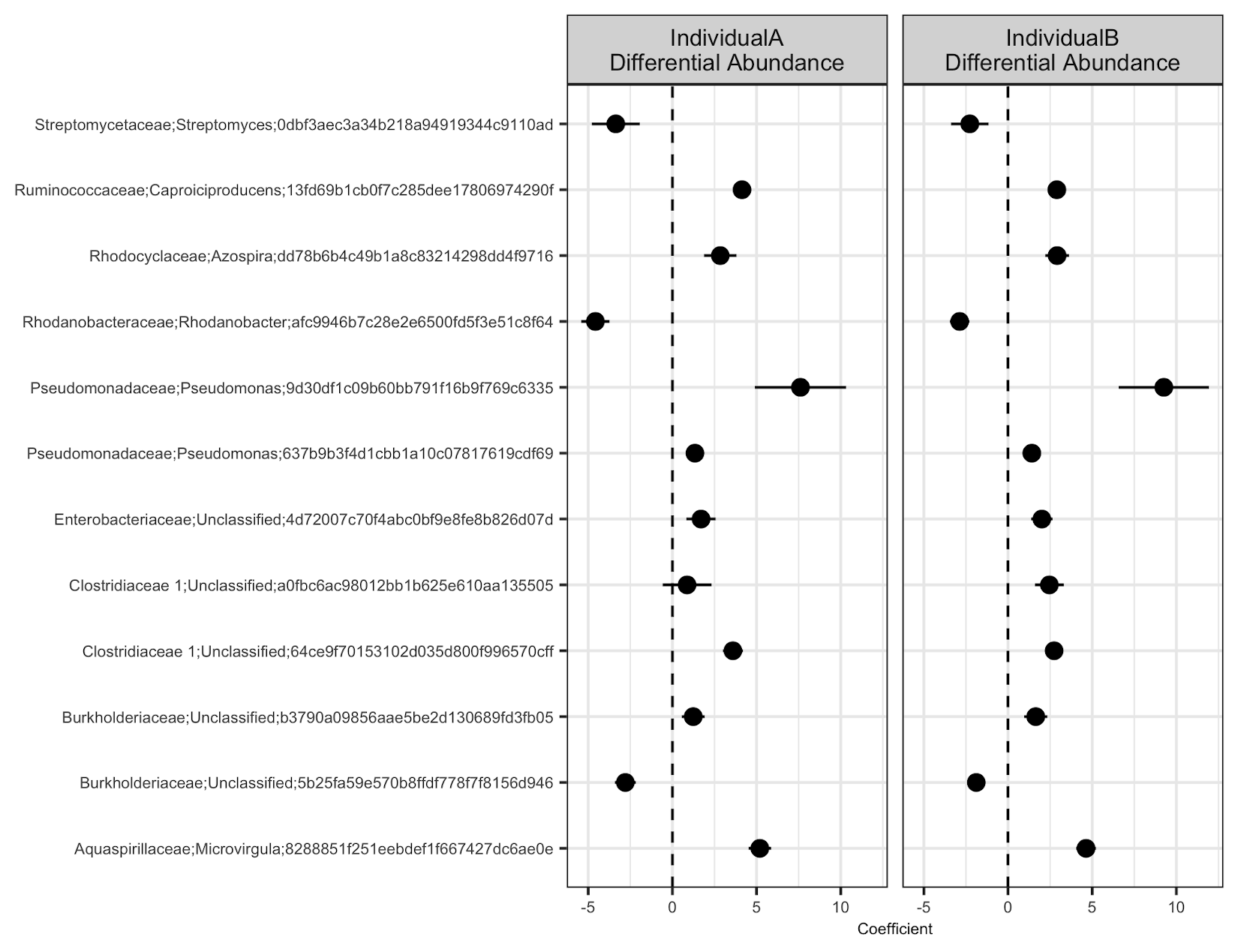

Supplement: FIG S5 [file msystems.00041-22-sf005.tif]
