# Supplementary material for: Postmortem Skeletal Microbial Community Composition and Function in Buried Human Remains
Source: mSystems. 2022 Mar 30;7(2):e00041-22. doi: 10.1128/msystems.00041-22 (PMC9040591; doi:10.1128/msystems.00041-22)
Supplement: TABLE S4 [file msystems.00041-22-st004.docx]

| Measure | Individual | Mean | Std. Dev. | Max. | Min. |
| --- | --- | --- | --- | --- | --- |
| Richness  (Observed ASVs) | AmerGut | 65.20 | 27.62 | 118.2 | 10.79 |
|  | A | 234.33 | 166.90 | 638.24 | 26.15 |
|  | B | 315.98 | 150.14 | 730.6 | 74.1 |
|  | C | 588.94 | 128.01 | 855.06 | 284.4 |
|  | SA | 324.02 | 120.86 | 595.88 | 88.05 |
|  | SB | 415.33 | 224.42 | 966.51 | 87.97 |
|  | SC | 388.93 | 116.17 | 683.82 | 205.34 |
|  | Soil | 600.07 | 159.20 | 890.07 | 354.88 |
| Diversity  (Inverse Simpson) | AmerGut | 7.70 | 7.46 | 24.77 | 1.03 |
|  | A | 20.26 | 21.74 | 114.76 | 1.96 |
|  | B | 19.91 | 16.19 | 89.67 | 1.65 |
|  | C | 46.16 | 25.59 | 119.86 | 5.22 |
|  | SA | 33.35 | 23.68 | 112.90 | 5.42 |
|  | SB | 48.82 | 40.21 | 199.06 | 3.32 |
|  | SC | 53.50 | 29.31 | 126.04 | 12.97 |
|  | Soil | 89.57 | 58.88 | 203.67 | 16.38 |
