# Supplementary material for: Postmortem Skeletal Microbial Community Composition and Function in Buried Human Remains
Source: mSystems. 2022 Mar 30;7(2):e00041-22. doi: 10.1128/msystems.00041-22 (PMC9040591; doi:10.1128/msystems.00041-22)
Supplement: TABLE S5 [file msystems.00041-22-st005.docx]

| Distance Metric | Test | Univariate/  Multivariate | *F* statistic | *R^2^* | *p* – value | Degrees of Freedom | Taxa  Level |
| --- | --- | --- | --- | --- | --- | --- | --- |
| Bray-Curtis  Weighted UniFrac | Project | Univariate | 40.5 | 0.15 | 0.001 | 2 | genus |
|  | Type | Univariate | 18.3 | 0.14 | 0.001 | 4 | genus |
|  | Surface / Subsurface bone | Univariate | 33.2 | 0.08 | 0.001 | 1 | genus |
|  | Individual (excludes AGP) | Univariate | 20.5 | 0.21 | 0.001 | 5 | genus |
|  | Individual (Grave) | Multivariate | 27.5 | 0.15 | 0.001 | 2 | ASV |
|  | Body Site (Grave) | Multivariate | 6.44 | 0.10 | 0.001 | 6 | ASV |
|  | Individual*Body Site | Multivariate | 4.87 | 0.16 | 0.001 | 12 | ASV |
|  | Project | Univariate | 57.8 | 0.20 | 0.001 | 2 | ASV |
|  | Type | Univariate | 27.6 | 0.20 | 0.001 | 4 | ASV |
|  | Surface / Subsurface Bones | Univariate | 40.2 | 0.10 | 0.001 | 1 | ASV |
|  | Individual (excludes AGP) | Univariate | 22.7 | 0.23 | 0.001 | 5 | ASV |
|  | Individual (Grave) | Multivariate | 35.6 | 0.18 | 0.001 | 2 | ASV |
|  | Body Site (Grave) | Multivariate | 7.48 | 0.12 | 0.001 | 6 | ASV |
|  | Individual*Body Site | Multivariate | 4.25 | 0.13 | 0.001 | 12 | ASV |
