# Supplementary material for: Postmortem Skeletal Microbial Community Composition and Function in Buried Human Remains
Source: mSystems. 2022 Mar 30;7(2):e00041-22. doi: 10.1128/msystems.00041-22 (PMC9040591; doi:10.1128/msystems.00041-22)
Supplement: TABLE S1 [file msystems.00041-22-st001.docx]

| Sample ID | Sample Type | Provenience | Depth (cm) |
| --- | --- | --- | --- |
| MGR2170021 | Soil | 0.5 m off grave | 0 |
| MGR2170023 | Soil | 0.5 m off grave | 30 |
| MGR2170037 | Soil | 0.5 m off grave | 0 |
| MGR2170039 | Soil | 0.5 m off grave | 30 |
| MGR2170053 | Soil | 0.5 m off grave | 0 |
| MGR2170055 | Soil | 0.5 m off grave | 30 |
| MGR2170005 | Soil | Control Grave | 0 |
| MGR2170007 | Soil | Control Grave | 30 |
| MGR2170001 | Soil | Control | 0 |
| MGR2170003 | Soil | Control | 30 |
| MGR2170057 | Soil | Grave | 0 |
| MGR2170059 | Soil | Grave | 0 |
| MGR3170002 | Soil | Grave | 0 |
| MGR3170003 | Soil | Grave | 0 |
| MGR3170004 | Soil | Grave | 0 |
| MGR3170005 | Soil | Grave | 0 |
| MGR3170006 | Soil | Grave | 30 |
| MGR3170007 | Soil | Grave | 40 |
| MGR3170008 | Soil | Grave | 30 |
| MGR3170009 | Soil | Grave | 30 |
| MGR3170026 | Soil | Grave | 70 |
| MGR3170027 | Soil | Grave | 85 |
| MGR3170028 | Soil | Grave | 70 |
| MGR3170029 | Soil | Grave | 85 |
| MGR3170030 | Soil | Grave | 70 |
| MGR3170031 | Soil | Grave | 70 |
| MGR3170032 | Soil | Grave | 85 |
