# Supplementary material for: Postmortem Skeletal Microbial Community Composition and Function in Buried Human Remains
Source: mSystems. 2022 Mar 30;7(2):e00041-22. doi: 10.1128/msystems.00041-22 (PMC9040591; doi:10.1128/msystems.00041-22)
Supplement: TABLE S2 [file msystems.00041-22-st002.docx]

| Body Region | Skeletal Element | Sample / Individual | Total DNA Samples |
| --- | --- | --- | --- |
| Skull | Frontal | 1 | 3 |
|  | Temporal | 1 | 3 |
|  | Maxilla | 1 | 3 |
|  | Parietal | 1 | 3 |
|  | Occipital | 1 | 3 |
|  | Mandible | 1 | 3 |
| Upper Torso | Cervical Vertebrae | 2 | 6 |
|  | 1st Rib | 2 | 6 |
|  | Middle Rib | 2 | 6 |
|  | Sternum | 1 | 3 |
|  | Clavicle | 2 | 6 |
|  | Scapula | 1 | 3 |
| Lower Torso | Thoracic Vertebrae | 1 | 3 |
|  | Lumbar Vertebrae | 1 | 3 |
|  | 11th or 12th Rib | 1 | 3 |
|  | Sacrum | 1 | 3 |
|  | Ilium | 1 | 3 |
|  | Ischium | 1 | 3 |
|  | Pubis | 1 | 3 |
| Leg | Femur | 6 | 18 |
|  | Tibia | 6 | 18 |
|  | Fibula | 1 | 3 |
|  | Patella | 2 | 6 |
| Arm | Humerus | 6 | 18 |
|  | Radius | 2 | 6 |
|  | Ulna | 2 | 6 |
| Hand | Metacarpal 1 | 1 | 3 |
|  | Metacarpal 2 | 1 | 3 |
|  | Metacarpal 3 | 1 | 3 |
|  | Metacarpal 4 | 2 | 6 |
|  | Metacarpal 5 | 1 | 3 |
|  | 1st Proximal Phalanx | 1 | 3 |
|  | 2nd Middle Phalanx | 1 | 3 |
|  | 1st Distal Phalanx | 2 | 6 |
|  | Capitate | 1 | 3 |
| Foot | Metatarsal 1 | 1 | 3 |
|  | Metatarsal 2 | 2 | 6 |
|  | Metatarsal 3 | 1 | 3 |
|  | Metatarsal 4 | 2 | 6 |
|  | Metatarsal 5 | 2 | 6 |
|  | 1st Proximal Phalanx | 1 | 3 |
|  | 1st Distal Phalanx | 1 | 3 |
|  | Calcaneus | 2 | 6 |
|  | Talus | 2 | 6 |
|  | Navicular | 2 | 6 |
|  | Cuboid | 2 | 6 |
|  | 1st Cuneiform | 2 | 6 |
|  | 2nd Cuneiform | 2 | 6 |
|  | 3rd Cuneiform | 2 | 6 |
