# Supplementary material for: Postmortem Skeletal Microbial Community Composition and Function in Buried Human Remains
Source: mSystems. 2022 Mar 30;7(2):e00041-22. doi: 10.1128/msystems.00041-22 (PMC9040591; doi:10.1128/msystems.00041-22)
Supplement: TABLE S3 [file msystems.00041-22-st003.docx]

| Prep ID | Sample ID | Age | Residence | weight (kg) | Sample Type |
| --- | --- | --- | --- | --- | --- |
| 1115 | 10317.000002261 | 52 | TN | 70 | Stool |
|  | 10317.000002262 | 54 | TN | 74 | Stool |
|  | 10317.000003439 | 50 | TN | 58 | Stool |
|  | 10317.000001812 | 56 | WA | 70 | Stool |
|  | 10317.000001830 | 58 | WA | 79 | Stool |
|  | 10317.000002211 | 69 | GA | 63 | Stool |
|  | 10317.000002219 | 61 | GA | 47 | Stool |
|  | 10317.000002220 | 66 | GA | 71 | Stool |
|  | 10317.000001194 | 60 | VA | 63 | Stool |
|  | 10317.000001826 | 68 | WA | 82 | Stool |
|  | 10317.000002884 | 60 | WA | 49 | Stool |
| 1116 | 10317.000001281 | 61 | TN | 78 | Stool |
|  | 10317.000001286 | 55 | TN | 52 | Stool |
|  | 10317.000004656 | 58 | GA | 46 | Stool |
|  | 10317.000001205 | 54 | VA | 76 | Stool |
|  | 10317.000001861 | 68 | VA | 72 | Stool |
|  | 10317.000002114 | 67 | VA | 126 | Stool |
|  | 10317.000004830 | 67 | WA | 125 | Stool |
|  | 10317.000004831 | 68 | WA | 144 | Stool |
|  | 10317.000005910 | 67 | WA | 60 | Stool |
| 1122 | 10317.000013020 | 67 | TN | 97 | Stool |
|  | 10317.000014979 | 52 | TN | 68 | Stool |
|  | 10317.000014111 | 56 | GA | 83 | Stool |
|  | 10317.000001187 | 57 | VA | 55 | Stool |
|  | 10317.000010744 | 57 | VA | 63 | Stool |
|  | 10317.000010909 | 59 | VA | 53 | Stool |
|  | 10317.000007023 | 60 | AL | 72 | Stool |
|  | 10317.000014974 | 69 | AL | 104 | Mouth |
|  | 10317.000002113 | 67 | VA | 124 | Stool |
|  | 10317.000003433 | 63 | VA | 113 | Stool |
|  | 10317.000009166 | 67 | VA | 96 | Stool |
|  | 10317.000013009 | 63 | WA | 88 | Stool |
| 1130 | 10317.000002259 | 52 | TN | 70 | Mouth |
|  | 10317.000009767 | 63 | TN | 28 | Stool |
|  | 10317.000003440 | 50 | TN | 58 | Mouth |
|  | 10317.000002111 | 57 | VA | 79 | Stool |
|  | 10317.000002112 | 58 | VA | 92 | Stool |
|  | 10317.000001811 | 55 | WA | 77 | Stool |
|  | 10317.000002221 | 60 | GA | 63 | Mouth |
|  | 10317.000002222 | 60 | GA | 63 | Stool |
|  | 10317.000002157 | 60 | VA | 79 | Stool |
|  | 10317.000006078 | 64 | VA | 56 | Stool |
|  | 10317.000007009 | 64 | VA | 97 | Stool |
|  | 10317.000007010 | 64 | VA | 97 | Mouth |
|  | 10317.000004651 | 67 | WA | 64 | Stool |
|  | 10317.000005702 | 68 | WA | 84 | Stool |
|  | 10317.000005703 | 68 | WA | 84 | Stool |
|  | 10317.000005911 | 67 | WA | 60 | Mouth |
|  | 10317.000006071 | 60 | WA | 56 | Stool |
| 1133 | 10317.000002260 | 52 | TN | 70 | Forehead |
|  | 10317.000009586 | 57 | TN | 60 | Stool |
|  | 10317.000003441 | 50 | TN | 58 | Right Hand |
|  | 10317.000009403 | 58 | GA | 70 | Stool |
|  | 10317.000009594 | 52 | VA | 53 | Stool |
|  | 10317.000003983 | 59 | WA | 104 | Stool |
|  | 10317.000005738 | 57 | WA | 85 | Stool |
|  | 10317.000007012 | 64 | VA | 97 | Hair |
| 1158 | 10317.000009768 | 63 | TN | 28 | Stool |
|  | 10317.000022443 | 52 | TN | 59 | Stool |
|  | 10317.000022444 | 55 | TN | 92 | Stool |
|  | 10317.000022520 | 51 | TN | 77 | Stool |
|  | 10317.000026437 | 57 | AL | 87 | Stool |
